# Supplementary material for: Perspectives of nurses’ role in interprofessional pharmaceutical care across 14 European countries: A qualitative study in pharmacists, physicians and nurses
Source: PLoS One. 2021 May 27;16(5):e0251982. doi: 10.1371/journal.pone.0251982 (PMC8158867; doi:10.1371/journal.pone.0251982)
Supplement: S1 Appendix — English version and 12 translations. (PDF) [file pone.0251982.s001.pdf]

<u>Interview guide for study "A SWOT analysis on the role of nurses' in multidisciplinary pharmaceutical care in Europe: a qualitative study"</u>

Instructions based on the discussions during the intensive study program, Antwerp, 2018:

- A short questionnaire (7 questions (demographics & job characteristics)) has to be completed before the interview.
- The model which was developed in advance, will not be shown to the informants.
- The informants will first be asked to reflect in a more open way on nurses' responsibilities, tasks and interactions. Afterwards, we will ask them to reflect on the things we had thought of before, using the specific contents of the model related to that question.
- The interviewer has to clearly state the definitions of the concepts used and has to guide the informant in understanding the questions and answering them within the scope.
- No documents can be send to the informants beforehand.
- All interviewers should be transparent about the selection of experts/ critical cases, applying the technique learned in the intensive study program.
- All data should be stored in a save way until the project coordinator explicitly states on paper that data can be removed.
- Any problems during data-collection or –analysis should be reported to the project coordinator.
- We advise you to write down the responsibilities, tasks and interactions reported by the informants in a structured way during the interview, as in topic 2 you reflect on all responsibilities reported by informants in topic 1, and in topic 4 you reflect on all responsibilities, tasks and interactions.

Introduction

- <u>Welcoming the participant:</u> "Hello, welcome. Thank you for being here today and to be willing to participate in this interview."
- <u>Describing the study:</u> "This interview is part of a large-scale European interview study in 14 countries about the role of nurses in multidisciplinary pharmaceutical care. Nurses, pharmacist and physicians of different health care settings will be interviewed. Our aim is to develop a general model for nurses' role in multidisciplinary pharmaceutical care, in order to improve multidisciplinary collaboration. We want you to think about what nurses' role in multidisciplinary pharmaceutical care would be like in an ideal situation. What would be ideal for pharmaceutical care? Afterwards we will ask you to reflect on the context of clinical practice and the prerequisites to implement this role. We will do this in a structured way asking for strengths and weaknesses of nurses' role today and about the opportunities and treats for the future. We will focus on the role of nurses, not the role of other healthcare professionals. There are no good or wrong answers on the questions. It's important that you give your personal opinion on the topic."
- "May I ask you, if possible, to switch off your mobile phone."
- <u>Explaining the course/procedure of the interview:</u> "This interview will be audiotaped. After the interview I will write out the interview and analyze the data without your name or other names that you might have mentioned during the interview. I will now start the audiotape. Is that OK for you?"
- <u>Asking for informed consen</u>t, give the participant the time to read the total document. "May I ask you to sign a form, that is called 'informed consent'? This means that you have been informed about the study, that you had enough time to ask questions, that you will take part voluntary and that you know you can terminate your participation at any time without giving any reason. After giving informed consent by signing the form, and

completing a short questionnaire with 7 questions about personal and job characteristics, I will start the interview, I will not call you by name during the interview, which will take 45-60min of your time. You can stop/pause the interview if you want so."

Topic 1

When we talk about pharmaceutical care, we want you to think about health care professionals contribution to the care of individuals in order to optimize medicines use and improve health outcomes.

In your opinion, looking at nurses' ideal role in multidisciplinary pharmaceutical care,

- What responsibilities would be part of the ideal role of a nurse? What do these responsibilities imply?
- When considering the next 4 responsibilities, what would you like to change, add or remove?

Responsibilities:
- monitoring & following-up adverse / therapeutic effects,<br>- monitoring & following-up medicines adherence,<br>- decision making on medicines use, including prescribing medicines, excluding preparation / administration, and<br>- providing patient education and information about medicines.

- **Topic 2**

Within these responsibilities, we also want to define the tasks nurses would ideally perform.

In your opinion, within the previously defined responsibilities of pharmaceutical care,

- Which specific tasks should nurses perform in their role in multidisciplinary pharmaceutical care?
- When considering the next tasks, what would you like to change, add or remove?

Tasks:
- Detecting clinical change, healthcare problems or assessing patient needs<br>- Registration<br>- Multidisciplinary communication (including reporting, alerting & discussion)<br>- Patient communication<br>- Intervention in case of emergency<br>- Follow-up<br>- Selfcare support<br>- 'Dependent' nurse prescribing<br>- 'Independent' nurse prescribing<br>- Reporting medication errors & safety issues

- **Topic 3**

High quality multidisciplinary pharmaceutical care requires agreements on the interaction between nurses, physicians and pharmacists. How can we best describe the ideal collaboration and communication between nurses and other health care professionals, aiming for high quality pharmaceutical care?

In your opinion, within the domain of pharmaceutical care,

- What do you think collaboration and communication between nurses and other health care providers in multidisciplinary pharmaceutical care should consist of?
- When considering the next interactions, what would you like to change, add or remove?

Interactions:<br>- Nurses reporting observations to physicians and pharmacists<br>- Physicians providing information and instructions to nurses<br>- Pharmacists giving advice to nurses

- **Topic 4**

We now have discussed on nurses' responsibilities, tasks and multidisciplinary interactions in multidisciplinary pharmaceutical care. We would like you to reflect now on the prerequisites to bring this ideal model to reality.

Please consider all responsibilities, tasks and multidisciplinary interactions.

1. What are strengths of nurses' role in multidisciplinary pharmaceutical care? What is going well already? What would you like to keep?
2. What are the weaknesses of nurses' role in multidisciplinary pharmaceutical care? What is not going well at the moment? What should be changed in nurses' role in aiming for high quality pharmaceutical care.
3. If we would like to bring the ideal model of nurses' role in multidisciplinary pharmaceutical care into practice, what are the opportunities? What makes it easier to realize the implementation? What are the favorable circumstances?
4. If we would like to bring the ideal model of nurses' role in multidisciplinary pharmaceutical care into practice, what are the threats? What makes it more difficult to realize the implementation? What are unfavorable circumstances?

<u>Translations of the interview guide</u>

- **Belgium + The Netherlands (1 language)**
- **Czech Republic**
- **Germany**
- **Greece**
- **Hungary**
- **Italy**
- **North Macedonia**
- **Norway**
- **Portugal**
- **Slovenia**
- **Slovakia**
- **Spain**

Interview guide

Belgium + The Netherlands

<u>Vragenprotocol voor de studie “Een SWOT analyse over de rol van verpleegkundigen in interdisciplinaire farmaceutische in Europa: een kwalitatief onderzoek.”</u>

Instructies gebaseerd op gesprekken tijdens het *Intensive Study Program* te Antwerpen in november 2018.

- Het vooraf ontwikkelde model wordt niet getoond aan de respondenten.
- Aan de respondenten worden eerst gevraagd om op een open en zo ruim mogelijke manier te reflecteren over de verantwoordelijkheden, taken en interacties van verpleegkundigen. Daarna vragen we hen te reflecteren over de zaken waarover we reeds ideeën vormden; er wordt daarbij gebruik gemaakt van de specifieke inhoud van het model waarover desbetreffende vraag handelt.
- De interviewer verheldert de gebruikte definities van het model en leidt de respondenten doorheen de vragen en antwoorden van het onderzoeksdoel.
- Er wordt op voorhand geen documenten overhandigd aan de respondenten.
- Alle interviewers dienen transparant te zijn in hun selectie van de beoogde experten; hierbij hanteren zij de technieken die aangereikt werden tijdens het *Intensive Study Program*.
- Alle data wordt opgeslagen in een beveiligde omgeving. Dit blijft zo totdat de projectcoördinator expliciet schriftelijk te kennen geeft dat de data verwijderd mag worden.
- Elk probleem dat opduikt tijdens de dataverzameling of -analyse wordt gemeld aan de projectcoördinator.
- Het is aangeraden de vermelde verantwoordelijkheden, taken en interacties door de respondenten te noteren tijdens de interviews. Zo kan er op gestructureerde wijze gereflecteerd worden; vb.: 'Bij thema 2 neem je al de verantwoordelijkheden in overweging die bij thema 1 aangehaald worden."

Inleiding

- <u>Begroeting</u>:
  "Dank u voor uw deelname aan dit interview."
- <u>Beschrijving van de studie</u>
  Dit interview is een onderdeel van een groot Europees kwalitatief onderzoek; de studie onderzoekt in 14 landen de rol van verpleegkundigen in interdisciplinaire farmaceutische zorg. Verpleegkundigen, artsen en apothekers uit verschillende werkomgevingen worden geïnterviewd. Ons doel is de ontwikkeling van een algemeen model voor de rol van verpleegkundigen in interdisciplinaire farmaceutische zorg. Met dit model willen we de samenwerking verbeteren.
  De vragen die gesteld worden hebben betrekking op de rol van verpleegkundigen; niet deze van apothekers en artsen. We willen u vragen na te denken over wat de verpleegkundige rol in de interdisciplinaire farmaceutische zorg is in de ideale situatie. Wat is *Ideale Farmaceutische Zorg*? Daarna vragen we u te reflecteren over de praktijkcontext en de voorwaarden die er moeten zijn om deze rol in de praktijk te brengen. Door te peilen naar sterktes en zwaktes van de huidige rol van de verpleegkundige wordt de vraagstelling op gestructureerde wijze ondersteund. Ook kansen en bedreigingen voor de toekomst komen hierbij aanbod. Er zijn geen juiste of foute antwoorden. Het is de bedoeling dat u uw persoonlijke mening geeft over dit onderwerp.
- "Is het mogelijk uw telefoontoestel uit of stil te zetten tijdens het interview?"
- <u>Uitleg over het verloop van het interview.</u>
  "Van dit gesprek worden geluidsopnames gemaakt. Na het interview schrijf ik het interview uit voor analyse; in de transcriptie wordt uw naam of deze van derden die mogelijk vermeld werden niet genoteerd. Ik start nu de geluidsopname. Is dat in orde voor u?"
- <u>Verzoek voor *Geïnformeerde toestemming*</u>
  Tijd geven aan de respondent om het hele document te lezen.
  "Ik zou u willen vragen het formulier *Geïnformeerde toestemming* te ondertekenen. Dit wil zeggen dat u inlichtingen heb gekregen over de studie, dat u voldoende tijd kreeg om vragen te stellen, dat u vrijwillig deelneemt en dat u op elk moment, zonder opgave van redenen, kan stoppen met het interview. Na het plaatsen van uw handtekening start ik het interview. Ik ga uw naam niet vernoemen tijdens het interview dat ongeveer 45 à 60 min duurt. U kan tijdens het gesprek stoppen of pauzeren."

Thema 1

Als we het hebben over farmaceutische zorg, dan willen we dat u nadenkt over ...(aanvullen!):
de bijdrage die gezondheidszorgprofessionals leveren aan de zorg voor individuen met het oog op het optimaliseren van medicatiegebruik en het verbeteren van gezondheidsuitkomsten.

Wat is uw idee over de ideale functie/rol/taak voor verpleegkundigen in interdisciplinaire farmaceutische zorg?

- Welke verantwoordelijkheden maken deel uit van deze ideale functie/rol/taak?
  Wat houden deze verantwoordelijkheden in?

Als u deze volgende vier verantwoordelijkheden ziet, wat zou u overwegen te veranderen, toe te voegen of verwijderen?

Verantwoordelijkheden:
- monitoren & opvolgen van therapeutische effecten en neveneffecten
- monitoren & opvolgen van therapietrouw
- beslissingen nemen over medicatiegebruik, inclusief voorschrijven van medicatie, met uitzondering van bereiden/toedienen
- geven van patiënteneducatie en informatie over geneesmiddelen

Thema 2

Binnen deze verantwoordelijkheden willen we de taken definiëren die verpleegkundigen idealiter moeten kunnen uitvoeren.

Wat is uw idee over de eerder vermelde verantwoordelijkheden in farmaceutische zorg?

- Welke specifieke taken zouden verpleegkundigen moeten uitvoeren in interdisciplinaire farmaceutische zorg binnenin de eerder beschreven verantwoordelijkheden?

Als u onderstaande taken in overweging neemt, wat zou u willen veranderen, toevoegen of verwijderen?

Taken
- Klinische verandering nagaan, gezondheidsklachten bevragen, behoeften van patiënten opsporen
- Registratie
- Interdisciplinaire communicatie (inclusief rapportage, waarschuwingen en discussie)
- Communicatie met patiënten
- Interventies in noodsituaties
- Opvolging
- Zelfzorg ondersteunen
- 'Afhankelijk' verpleegkundig voorschrijven
- 'Onafhankelijk' verpleegkundig voorschrijven
- Rapporteren van medicatiefouten en veiligheid-geassocieerde voorvallen

Thema 3

Voor hoogstaande kwalitatieve interdisciplinaire farmaceutische zorg zijn er afspraken nodig over de interactie en samenwerking tussen verpleegkundigen, artsen en apothekers.
Hoe kunnen we deze ideale samenwerking en communicatie tussen verpleegkundigen en andere disciplines in de gezondheidszorg het best beschrijven met als doel hoge kwalitatieve farmaceutische zorg?

Op vlak van farmaceutische zorg:

- Uit wat moet, volgens u, de samenwerking en communicatie bestaan tussen verpleegkundigen en zorgverleners in interdisciplinaire farmaceutische zorg?

Als u onderstaande interacties in overweging neemt, wat zou u willen veranderen, toevoegen of verwijderen?

Interacties:
- Verpleegkundigen rapporteren observaties aan artsen en apothekers
- Artsen bezorgen verpleegkundigen informatie en instructies
- Apothekers geven advies aan verpleegkundigen

<u>Thema 4</u>

We hebben het nu gehad over de verantwoordelijkheden van verpleegkundigen, hun taken en de interdisciplinaire interacties in interdisciplinaire farmaceutische zorg.
Nu willen we graag dat u uw mening geeft over de voorwaarden die dit ideale model in de praktijk kan doen werken.

Neem alle verantwoordelijkheden, taken en interdisciplinaire interacties in overweging.

1. Wat zijn sterke punten in de verpleegkundige rol in interdisciplinaire farmaceutische zorg?
   Wat gaat er nu al goed?
   Wat wil je behouden?
2. Wat zijn zwakke punten in de verpleegkundige rol in interdisciplinaire farmaceutische zorg?
   Wat gaat er nu niet goed?
   Wat zou er moeten veranderen in de verpleegkundige rol om hoogstaande farmaceutische zorg te verkrijgen?
3. Als we het *Ideale Model* voor de verpleegkundige rol in interdisciplinaire farmaceutische zorg in de praktijk willen brengen, waar/wat zijn dan de mogelijkheden?
   Hoe zou men het *Ideale Model* gemakkelijker kunnen implementeren?
   Zijn er bevorderende omstandigheden?
4. Als we het *Ideale Model* voor de verpleegkundige rol in interdisciplinaire farmaceutische zorg in de praktijk willen brengen, waar situeren zich de bedreigingen?
   Wat zou de implementatie kunnen bemoeilijken?
   Wat zijn belemmerende omstandigheden?

Interview guide

Czech Republic

Interview guide Cz

A SWOT analysis on the role of nurses’ in interprofessional pharmaceutical care in Europe:
a qualitative study

- **Organizační záležitosti:**
  - Vlastnímu rozhovoru bude předcházet krátký **dotazník** (demografické údaje), bude vyplněn s podpisem informovaného souhlasu
  - Předběžný návrh modelu nebude nejprve prezentován
  - Účastníci budou vždy nejprve požádáni, aby sami reflektovali a vyjádřili názory na oblasti zodpovědnosti sester, jejich činnosti a způsob interakce v rámci týmu
  - Následně budou požádáni o vyjádření názoru na předběžný model
  - Tazatel musí být schopen jasně definovat používané výrazy a dostatečně přiblížit a vysvětlit problematiku řešenou v rámci rozhovoru
  - Informace nebudou zasílány účastníkům předem
  - Je potřeba zajistit transparentní způsob výběru expertů (účastníků)
  - Veškerá data bude zapotřebí bezpečně uchovat, dokud nebude rozhodnuto o jejich skartaci.
  - Jakékoli problémy při sběru dat nebo analýze je potřeba hlásit koordinátorovi projektu.
  - Předběžný seznam zodpovědností, úkolů a interakcí bude mít tazatel připraveny v písemné podobě pro potřeby podrobnější diskuse.

- **Úvod**
  - Přivítání účastníka
    - Děkuji, že jste souhlasil/a s participaci na tomto projektu
  - Popis studie
    - Rozhovor je součásti velké mezinárodní studie, která je prováděna ve 14 zemích a týká se role sestry v rámci multidisciplinárním týmu při poskytování farmaceutické péče a podávání léčivých přípravků
    - A to z pohledu sester samotných, lékařů a farmaceutů působících v různých oblastech zdravotní péče
    - Rádi bychom popsali způsob spolupráce v rámci multidisciplinárního týmu v oblasti farmaceutické péče a podávání léčivých přípravků v současné době, a zaměřili se především na roli sestry
    - A následně vytvořit model (role sestry), který by vycházel z potřeb současné praxe, bylo by jej možné využít pro vzdělávání sester, s cílem přispět k optimalizaci (a tím zlepšení) péče v této oblasti a zároveň poskytnout sestrám určitou oporu tak, aby byly schopny na požadavky praxe reagovat.
    - Rádi bychom, abyste se zkusil/a zamyslet na tom, jak by v ideálním situaci měla vypadat role sestry v této oblasti
    - Jak by v ideálním případě měla péče v této oblasti vypadat?
    - Za jakých podmínek by bylo možné, aby tato péče takto vypadala?
    - Za jakých podmínek by ji bylo možné takto implementovat?
    - A následně, abyste se pokusil/a o reflexi současného stavu?
    - Postupně Vás poprosím, abyste se zamyslel/a také nad silnými a slabými stránkami v současné době a nad případnými příležitostmi a hrozbami, které je možné očekávat v budoucnu
    - Přestože se jedná o spolupráci v rámci multidisciplinárního týmu, rádi bychom se zaměřili na roli sestry, nikoli roli dalších zdravotnických pracovníků
    - Žádná odpověď na naše otázky není dobrá nebo špatná (správná nebo chybná), všechny odpovědi a zkušenosti jsou pro nás cenné (nejde o většinový názor)
    - Je pro nás důležité slyšet Váš osobní názor na dané téma
  - Můžu poprosit, abyste vypnul/a mobilní telefon?
  - Organizace rozhovoru
    - Rozhovory budou nahrávány. Následně přepsány. V přepisu, ale určitě nebudou uvedeny žádné osobní údaje nebo jména, která byste případně během rozhovoru uvedl/a

Interview guide Cz

A SWOT analysis on the role of nurses' in interprofessional pharmaceutical care in Europe:
a qualitative study

- **Teď si dovolím zahájit nahrávání**
- Informovaný souhlas
  - Teď Vás ještě poprosím o podepsání informovaného souhlasu
  - Během rozhovoru Vás nebudu oslovovat jménem
  - Předpokládám, že rozhovor by měl trvat asi 45-60 minut.
  - Rozhovor je samozřejmě možné kdykoli přerušit nebo ukončit, pokud se tak rozhodnete.

- **Téma 1**

  Pokud budeme mluvit o farmakoterapeutické péči, máme na mysli participaci různých zdravotnických pracovníků při poskytování zdravotní péče s cílem optimálního použití LP v péči o zdraví.
  - Když se zamyslíte nad tím, jak by měla ideálně vypadat role sestry při podávání LP v rámci multidisciplinární péče
    - Co by mělo být v ideálním případě náplní práce sestry, jaké činnosti by měla vykonávat, za co by měla zodpovídat ...
    - Co Vás napadá při pohledu na tyto 4 oblasti, je něco, co byste změnil/a, přidala, nebo naopak odstranila?

Zodpovědnost a role při:
- monitoraci & sledování nežádoucích/terapeutických účinků,
- monitoraci & sledování dodržování terapeutického režimu,
- ordinaci LP, s výjimkou přípravy/podání LP, včetně preskripce,
- edukaci pacienta & podávání informací o LP.

- **Téma 2**

  Rádi bychom také blíže určili činnosti, které by měla sestra ideálně v rámci své zodpovědnosti v těchto oblastech vykonávat.
  - Podle Vašeho názoru, pokud budeme uvažovat tyto oblasti zodpovědnosti ….
    - Které konkrétní činnosti by měly být náplni sestry v těchto uvedených oblastech její zodpovědnosti?
    - Pokud se podíváte na tyto navrhované činnosti, co Vás napadá, co byste změnil/a, přidal/a, odstranil/a…?

Činnosti:
- Identifikace změn zdravotního stavu, obtíží a potřeb pacienta
- Záznam do zdravotnické dokumentace
- Mezioborová komunikace (včetně dokumentace, hlášení & diskuze)
- Komunikace s pacientem
- Intervence u neodkladných stavů
- Dlouhodobé sledování
- Podpora sebepéče
- 'Závislá' preskripce (LP indikovány lékařem)
- 'Nezávislá' preskripce (některé LP indikovány sestrou)
- Hlášení nežádoucích příhod
- Supervize ostatních zdravotnických pracovníků

- **Téma 3**

  Kvalitní multidisciplinární péče klade také požadavky na vzájemnou spolupráci a interakci jednotlivých členů multidisciplinárního týmů, tj. na spolupráci sester, lékařů a farmaceutů. Jak by bylo možno popsat ideální způsob spolupráce těchto profesí s cílem dosáhnout co nejkvalitněji poskytované péče?
  - Podle Vašeho názoru
    - Jak by měla spolupráce a komunikace vypadat, co všechno by měla zahrnovat?
    - Pokud bychom uvažovali tento návrh, co něco, co byste rád/a změnila, přidala, s čím nesouhlasíte...?

Interakce:
- Sestra hlásí lékaři a farmaceutovi výsledky jejího pozorování (a hodnocení stavu)
- Lékař poskytuje sestře informace a pokyny
- Farmaceut poskytuje sestře doporučení

- **Téma 4**
  - Teď prosím, uvažujme všechny zmiňované oblasti zodpovědnosti sestry, všechny činnosti a způsoby interakce v rámci multidisciplinárního týmu, co by byly podmínky, předpoklady úspěšného zavedení ideálního modelu role sestry do praxe?
  - Prosím, uvažujte **všechny oblasti** zodpovědnosti sestry (o kterých jsme hovořili), všechny činnosti a způsoby interakce v rámci multidisciplinárního týmu
    1) Co by bylo možné považovat za silné stránky role sestry v současné době
       - Co už v současné době funguje dobře?
       - Co by mělo takto zůstat?
    2) A teď co byste naopak považoval/a za slabiny?
       - Co není v současné době řešeno optimálně?
       - Co byste považoval/a za důležité změnit, zlepšit, řešit jinak …?
    3) Pokud bychom chtěli realizovat a uvést do praxe ideální podobu multidisciplinární farmaceutické péče, jaké okolnosti je možné považovat za příležitost?
       - Čeho by bylo možné využít pro úspěšné uvedení tohoto modelu do praxe?
       - Co by bylo možné považovat za příznivé okolnosti, které by implementaci usnadnily?
    4) A naopak, co by bylo potřeba považovat za hrozbu úspěšné implementace tohoto modelu do praxe?
       - Díky čemu by mohla být realizace tohoto záměru obtížná/neproveditelná?
       - Co by bylo možné považovat za nepříznivé okolnosti?

Interview guide

Germany

Interviewleitfaden für die Studie „Eine SWOT-Analyse der Rolle von Pflegekräften in der multidisziplinären pharmazeutischen Betreuung in Europa: eine Interviewstudie“

Einführung:

- Begrüßung der Teilnehmerinnen und Teilnehmer: „Guten Tag, willkommen. Danke, dass Sie da sind und sich bereit erklärt haben das Interview mit mir zu führen.“
- Studienbeschreibung: „Dieses Interview ist Teil einer großen europäischen Interviewstudie mit 14 beteiligten Ländern, welche die Rolle von Pflegekräften in der multiprofessionellen pharmazeutischen Betreuung untersucht. Pflegende, Apotheker und Apothekerinnen sowie Ärzte und Ärztinnen der verschiedenen Arbeitsbereiche der Gesundheitsversorgung werden befragt. Das Ziel ist es ein allgemeines Modell der Rolle von Pflegekräften in der multiprofessionellen pharmazeutischen Betreuung zu generieren um die interdisziplinäre Zusammenarbeit zu verbessern. Wir würden gern von Ihnen wissen wie die Rolle von Pflegekräften in der multiprofessionellen pharmazeutischen Betreuung idealer Weise aussehen würde. Was wäre ideal für die pharmakologische Betreuung? Nachfolgend werden wir Sie bitten, die klinische Praxis und die Voraussetzungen dieser Rolle diesbezüglich zu reflektieren. Dies wird strukturiert ablaufen. Es geht um, die Stärken und Schwächen der Rolle einer Pflegekraft heute, sowie die Möglichkeiten und Risiken in Zukunft. Der Fokus liegt auf der Rolle Pflegender, nicht die anderen medizinischen Fachkräfte. Es gibt keine falschen oder richtigen Antworten auf die Fragen. Wichtig ist uns Ihre Meinung zu diesem Thema zu erfahren.“
- „Ich möchte Sie gern bitten Ihr Handy abzuschalten.“
- Das Vorgehen des Interviews erklären: „Das Interview wird aufgenommen. Anschließend wird das Interview transkribiert und analysiert, dabei werden sowohl Ihr Name als auch im Interview genannte weitere Namen nicht genannt werden. Wenn es für Sie in Ordnung ist würde ich jetzt die Aufnahme starten.“
- Nach Informed Consent fragen: Den Teilnehmern ausreichend Zeit zum Lesen des gesamten Dokuments geben. „Ich möchte Sie nun bitten die Einwilligungserklärung zu unterscheiben. Dies bedeutet, dass Sie über die Studie informiert wurden, Sie Rückfragen stellen konnten, Sie freiwillig teilnehmen und Sie wissen, dass Sie jederzeit Ihre Teilnahme ohne Angabe von Gründen zurückziehen können. Nachdem Sie die Einwilligungserklärung unterschrieben haben, werde ich mit dem Interview beginnen. Das Interview dauert zwischen 45 und 60 Minuten und währenddessen werde ich Sie nicht mit Namen ansprechen. Sie können das Interview abbrechen oder pausieren, wann immer sie möchten.

Themenbereich 1

Wenn wir über pharmazeutische Begleitung sprechen, möchten wir ihr Augenmerk auf folgendes lenken…

Ihrer Meinung nach, in Hinblick auf eine ideale Rolle von Pflegekräften im Rahmen der pharmazeutischen Betreuung,

- Welche Verantwortungsbereiche wären Teil einer solchen beruflichen Rolle einer Pflegekraft? Was würden diese beinhalten?
- Wenn Sie die nachfolgenden Verantwortungsbereiche betrachten, welchen würden Sie anpassen, ergänzen oder verwerfen?

Verantwortungsbereich:

- Überwachung und Nachverfolgung von unerwünschten/therapeutischen Wirkungen,

- Überwachung und Nachverfolgung der Einhaltung der Medikamenteneinnahme,
- Entscheidung über den Einsatz von Medikamenten, einschließlich der Verschreibung von Medikamenten, jedoch ohne Zubereitung/Verwaltung, und
- Patientenaufklärung und Informationsweitergabe über Arzneimittel.

Themenbereich 2

Im Zusammenhang mit den Verantwortungsbereichen möchten wir gern die Aufgaben definieren, welche Pflegekräfte idealerweise bei diesen erfüllen würden.

Innerhalb der eben definierten Aufgabenbereiche der pharmazeutischen Betreuung wie würden Sie:

- Die genauen Aufgaben von Pflegekräften in ihrer Rolle in der multidisziplinären pharmazeutischen Betreuung beschreiben?
- Wenn Sie die nachfolgenden Aufgaben betrachten, welche würden Sie anpassen, ergänzen oder verwerfen?

<u>Aufgaben:</u>

- Erkennen von Veränderungen des klinischen Zustandes, von Gesundheitsproblemen oder Erfassung der Patientenbedürfnisse

- Dokumentation

- Multidisziplinäre Kommunikation (inklusive berichten, alarmieren und Diskussion)

- Patientenkommunikation

- Intervention im Notfall

- Follow-up

- Unterstützung bei der Selbstfürsorge

- „abhängige“ Medikamentenverschreibung durch Pflegekraft - „unabhängige“ Medikamentenverschreibung durch Pflegekraft

- Meldung von Medikationsfehlern und Sicherheitsrisiken

Themenbereich 3

Eine sehr gute multidisziplinäre pharmazeutische Betreuung benötigt Übereinkünfte zwischen Pflegekräften, Ärzten und Ärztinnen und Apothekern und Apothekerinnen in Bezug auf die Zusammenarbeit. Wie würde die ideale Zusammenarbeit Kooperation und Kommunikation zwischen Pflegekräften und anderen Gesundheitsfachberufen mit dem Ziel einer qualitativ hochwertigen pharmazeutischen Betreuung aussehen?

Im Rahmen der pharmazeutischen Betreuung:

- Wie sollte ihrer Meinung nach die Zusammenarbeit und Kommunikation zwischen Pflegekräften und anderen Gesundheitsfachberufen in Bezug auf die multidisziplinäre pharmazeutische Betreuung aussehen/gestaltet werden?
- Wenn Sie die nachfolgenden Aspekte der Zusammenarbeit betrachten, welche würden sie anpassen, ergänzen oder verwerfen?

<u>Zusammenarbeit:</u>

- Pflegekräfte berichten ihre Beobachtungen gegenüber dem ärztlichen Personal und Apothekerinnen und Apothekern

- Ärzte und Ärztinnen versorgen Pflegekräfte mit Informationen und Instruktionen

- Apotheker und Apothekerinnen stehen den Pflegekräften beratend zur Seite

Kommentiert [KT1]: Diese beiden Begriffe müssten sicher erläutert werden, oder?

Themenbereich 4

Vorhergehend wurden die Verantwortungsbereiche, Aufgaben und die Zusammenarbeit von Pflegekräften in der multidisziplinären pharmazeutischen Betreuung besprochen. Wir möchten Sie nun bitten, die Voraussetzungen zur Realisierung dieses Idealmodells zu reflektieren /bedenken.

Bitte beachten Sie dabei alle Verantwortungsbereiche, Aufgaben und multidisziplinären Interaktionen.

1. Welche Stärken sehen Sie in der Rolle von Pflegekräften innerhalb der multidisziplinären pharmazeutischen Betreuung? Was läuft bisher gut? Was würden Sie beibehalten wollen?

2. Welche Nachteile (Schwierigkeiten?) sehen Sie in der Rolle von Pflegekräften innerhalb der multidisziplinären pharmazeutischen Betreuung? Welche Probleme sehen Sie im Moment? Wie sollte sich die Rolle der Pflegenden ändern um eine hochwertige pharmazeutische Betreuung bereitzustellen?

3. Bei einer Einführung des Idealmodells der Rolle von Pflegenden in der multidisziplinären pharmazeutischen Betreuung, welche Optionen gäbe es? Was könnte die Implementierung vereinfachen? Welche begünstigenden Umstände gibt es?

4. Bei einer Einführung des Idealmodells der Rolle von Pflegenden in der multidisziplinären pharmazeutischen Betreuung, welche Herausforderungen gäbe es? Welche Schwierigkeiten könnten sich bei der Implementierung ergeben? Welche hindernden Umstände gäbe es?

Interview guide

Greece

Interview guide DeMoPhaC - Greece

Μέρος 1ο

Ποιες αρμοδιότητες θα αποτελούσαν μέρος του ιδανικού ρόλου ενός νοσηλευτή στη δι- επαγγελματική φαρμακευτική φροντίδα; Τι συνεπάγονται/ πώς ερμηνεύονται αυτές οι αρμοδιότητες;

Εξετάζοντας τις επόμενες 4 αρμοδιότητες, τι θα θέλατε να αλλάξετε, να προσθέσετε ή να αφαιρέσετε;

Αρμοδιότητες:

- ⇨ Καταγραφή και παρακολούθηση ανεπιθύμητων ενεργειών/ θεραπευτικών αποτελεσμάτων – αποτελεσμάτων της θεραπείας
- ⇨ Καταγραφή και παρακολούθηση της συμμόρφωσης στη φαρμακευτική αγωγή,
- ⇨ Λήψη αποφάσεων σχετικά με τη χρήση της φαρμακευτικής αγωγής, συμπεριλαμβανομένης της συνταγογράφησης φαρμάκων και εξαιρουμένης της προετοιμασίας /χορήγησης αυτών, και
- ⇨ Εκπαίδευση και ενημέρωση ασθενών σχετικά με τη φαρμακευτική αγωγή

Μέρος 2ο

Στο πλαίσιο των αρμοδιοτήτων που προσδιορίσαμε προηγουμένως σχετικά με τη φαρμακευτική φροντίδα, θα θέλαμε τώρα να προσδιορίσουμε τα καθήκοντα που ιδανικά θα αναλάμβαναν οι νοσηλευτές.

Ποια συγκεκριμένα καθήκοντα πρέπει να εκτελούν οι νοσηλευτές, σαν μέρος του ρόλου τους, στη δι- επαγγελματική φαρμακευτική φροντίδα; (για κάθε αρμοδιότητα ξεχωριστά)

Εξετάζοντας τα καθήκοντα που θα σας αναφέρω, τι θα θέλατε να αλλάξετε, να προσθέσετε ή να αφαιρέσετε;

Καθήκοντα:

- ⇨ Ανίχνευση κλινικών αλλαγών, προβλημάτων υγειονομικής περίθαλψης ή αξιολόγηση των αναγκών του ασθενή
- ⇨ Εγγραφή
- ⇨ Δι- επαγγελματική επικοινωνία (περιλαμβανομένης της αναφοράς, της ειδοποίησης και της συζήτησης)
- ⇨ Επικοινωνία με τους ασθενείς
- ⇨ Παρέμβαση σε περίπτωση έκτακτης ανάγκης
- ⇨ Παρακολούθηση
- ⇨ Υποστήριξη αυτό- φροντίδας
- ⇨ "Εξαρτημένη" νοσηλευτική συνταγογράφηση
- ⇨ "Ανεξάρτητη" νοσηλευτική συνταγογράφηση
- ⇨ Αναφορά λαθών στη φαρμακευτική αγωγή και θεμάτων ασφάλειας

Μέρος 3ο

Να περάσουμε στο κομμάτι της αλληλεπίδρασης μεταξύ των Νοσηλευτών, των Ιατρών και των Φαρμακοποιών. Για την παροχή υψηλής ποιότητας, δι- επαγγελματικής φαρμακευτικής φροντίδας απαιτείται η από κοινού αποδοχή της μορφής/τρόπου αλληλεπίδρασης μεταξύ των Νοσηλευτών, των Ιατρών και των Φαρμακοποιών.

Πώς θα μπορούσαμε να περιγράψουμε καλύτερα την ιδανική συνεργασία και επικοινωνία μεταξύ των νοσηλευτών και των άλλων επαγγελματιών υγείας, στοχεύοντας στην υψηλής ποιότητας φαρμακευτική φροντίδα;

Κατά τη γνώμη σας, στο πλαίσιο της φαρμακευτικής φροντίδας,

Από ποια στοιχεία πρέπει να συνίσταται η συνεργασία και η επικοινωνία μεταξύ των νοσηλευτών και των άλλων επαγγελματιών υγείας, στο πλαίσιο της δι-επαγγελματικής φαρμακευτικής φροντίδας;

Εξετάζοντας τις ακόλουθες αλληλεπιδράσεις, τι θα θέλατε να αλλάξετε, να προσθέσετε ή να αφαιρέσετε;

Αλληλεπιδράσεις:

- ⇨ Οι Νοσηλευτές αναφέρουν τις παρατηρήσεις τους στους Ιατρούς και στους Φαρμακοποιούς
- ⇨ Οι Ιατροί παρέχουν πληροφορίες και οδηγίες στους Νοσηλευτές
- ⇨ Οι φαρμακοποιοί συμβουλεύουν τους Νοσηλευτές

Μέρος 4ο

Τώρα θα μεταφερθούμε στο πλαίσιο της κλινικής πρακτικής και θα σχολιάσουμε τις προϋποθέσεις που απαιτούνται για την εφαρμογή αυτού του νοσηλευτικού ρόλου, σχολιάζοντας τα δυνατά και τα αδύναμα σημεία του νοσηλευτικού ρόλου σήμερα, καθώς και τις ευκαιρίες και τις απειλές του βλέπετε στο μέλλον. Θα θέλαμε να σχολιάσετε πάνω στις προϋποθέσεις που θα πρέπει να πληρούνται ώστε να εφαρμοστεί αυτό το ιδανικό μοντέλο στην πράξη.

Παρακαλώ σκεφτείτε όλες τις αρμοδιότητες, τα καθήκοντα και τις δι- επαγγελματικές αλληλεπιδράσεις.

1. Ποια πιστεύετε ότι είναι σήμερα τα πλεονεκτήματα/δυνατά σημεία του ρόλου των νοσηλευτών στη δι- επαγγελματική φαρμακευτική φροντίδα; Τι λειτουργεί ήδη σωστά; Τι θα θέλατε να κρατήσετε;

2. Ποιά είναι τα αδύναμα σημεία του ρόλου των νοσηλευτών στη δι-επαγγελματική φαρμακευτική φροντίδα σήμερα; Τι δεν λειτουργεί σωστά στο παρόν; Τι θα πρέπει να αλλάξει όσον αφορά το ρόλο των νοσηλευτών, στοχεύοντας στην υψηλής ποιότητας φαρμακευτική φροντίδα;

3. Εάν θέλαμε να εφαρμόσουμε στην πράξη αυτό το ιδανικό μοντέλο του ρόλου των νοσηλευτών στη δι- επαγγελματική φαρμακευτική φροντίδα, ποιες θεωρείτε ότι είναι οι ευκαιρίες για να γίνει αυτό; Τι διευκολύνει την υλοποίηση της εφαρμογής; Ποιες συνθήκες την ευνοούν;

4. Εάν θέλαμε να εφαρμόσουμε στην πράξη αυτό το ιδανικό μοντέλο του ρόλου των νοσηλευτών στη δι- επαγγελματική φαρμακευτική φροντίδα, ποιες θεωρείτε ότι είναι οι απειλές; Τι δυσχεραίνει την υλοποίηση της εφαρμογής; Ποιες συνθήκες/καταστάσεις δρουν ανασταλτικά;

Interview guide

Hungary

Interjú útmutató a kutatáshoz “Egy SWOT elemzés az ápolók szerepéről a multidiszciplináris gyógyszerészeti ellátásban Európában: kvalitatív kutatás”

Instrukciók a 2018-as antwerpeni ISP-n megbeszéltek alapján:

• Az előzetesen kifejlesztett modellt nem fogjuk bemutatni az interjúk alanyainak.

• Az alanyok először beszéljenek nem irányított módon az ápolók szerepéről, felelősségeiről, kapcsolatairól más szakmákkal. Ezután, megkérjük őket, hogy reagáljanak azokra a pontokra, melyeket előzetesen meghatároztunk, felhasználva a modell specifikus összetevőit, melyek a kérdéshez kapcsolódnak.

• Az interjú készítőjének világosan meg kell határoznia az interjú során használt fogalmakat, biztosítania kell, hogy az alany érti a kérdéseket és az interjút megfelelő mederben kell tartania.

• Semmi féle dokumentum nem küldhető el előzetesen az alanynak.

• Az összes kutatónak készen kell állnia kérdésekre az alanyok választásának menetével kapcsolatosan és azokra kimerítően válaszolniuk kell.

• Az összes adatot biztonságosan kell tárolni mindaddig, amíg a projekt vezetője írásbeli felhatalmazást nem ad azok törlésére.

• Bármilyen probléma az adatgyűjtéssel, és -elemzéssel kapcsolatosan jelentendő a projektvezetőnek.

• Javasoljuk, hogy az interjúk során írd le az alany által említett felelősségeket, feladatokat, interakciókat strukturált módon, így például a kettes témakörben reflektálhatsz az alany által az egyes témakörön belül megemlített felelősségekre, a négyes témakörben pedig az összes felelősségre, feladatra, interakcióra.

Bemutatás

• <u>A résztvevő üdvözlése</u>: “Üdvözlöm. Köszönöm, hogy eljött és hajlandó részt venni az interjún.”

• <u>A kutatás bemutatása</u>: “Ez az interjú egy nagyszabású európai szintű kutatás részét képezi, mely 14 országban zajlik és az ápolók szerepét kutatja az interdiszciplináris gyógyszerészeti ellátásban. Ápolók, gyógyszerészek és orvosok vesznek részt az interjúkban, különböző egészségügyi ellátási területekről. Célunk egy általános modell kifejlesztése ápolók számára a gyógyszerészeti ellátásra vonatkozóan a multidiszciplináris együttműködés fejlesztése érdekében. Azt szeretnénk, ha elgondolkodna azon, hogy ideális helyzetben mi lenne az ápolók szerepe a multidiszciplináris gyógyszerészeti ellátásban. Mi lenne a legjobb az ellátás fejlődése érdekében? Ezután, arra kérjük, hogy beszéljen a klinikai gyakorlati valóságról és vegye számba, milyen előfeltételek lennének szükségesek a kívánt változások elérése érdekében. Ezt strukturált módon tesszük, megkérjük majd, hogy beszéljen az erősségekről és gyengeségekről az ápolók jelenlegi szerepével kapcsolatosan és a jövőbeni lehetőségekről és veszélyekről is. A fókuszt az interjú során az ápolók szerepére helyezzük, nem egyéb egészségügyi dolgozók szerepére. Nincsenek jó vagy rossz válaszok, fontos, hogy a saját véleményét fejezze ki a témával kapcsolatosan.”

• “Amennyiben lehetséges, kérem kapcsolja ki a mobiltelefonját.”

• Az interjú menetének elmagyarázása: “Az interjúról hangfelvétel készül. Az interjút követően le fogom gépelni a beszélgetésünk szövegét és elemezni fogom az adatokat név nélkül. Ha ez így rendben van, akkor most elindítom a hangfelvételt.”

• A beleegyező nyilatkozat aláíratása: adjunk a résztvevőnek elég időt a nyilatkozat átolvasására.

„ Megkérhetem, hogy olvassa el és írja alá a beleegyező nyilatkozatot? Ezzel kijelenti, hogy megfelelően tájékoztattuk a kutatásról, volt ideje kérdéseket feltenni, önkéntesen vesz részt a kutatásban és tudja, hogy bármikor elállhat a részvételtől indoklás nélkül. Miután aláírta a nyilatkozatot, megkezdhetjük az interjút. Az interjú során, mely kb 45-60 percet vesz igénybe, nem fogom a nevén szólítani. Ha kéri, tarthatunk szünetet is közben.”

Első témakör

Amikor a gyógyszerészeti ellátásról beszélünk, az egészségügyi dolgozók hozzájárulását értjük alatta az egyes páciensek gyógyszerelésének optimalizálása és végső soron az egészségügyi kimenetel fejlesztése érdekében.

Véleménye szerint, az ápolók ideális szerepét tekintve a gyógyszerészeti ellátásban,

• Mely felelősségek képeznék részét az ápolók szerepkörének? Mi mindent foglalnak magukba ezek a felelősségek?

•Az alábbi négy felelősséget vizsgálva miket változtatna meg, távolítana el vagy adna hozzá?

- A terápiás-, illetve mellékhatások megfigyelése és követése.

- A gyógyszeres kezelés betartásának megfigyelése és követése.

- Döntéshozatal a gyógyszereléssel kapcsolatosan, beleértve a gyógyszerfelírást, nem beleértve az előkészítést és gyógyszerbeadást.

- Betegedukáció és a gyógyszerekkel kapcsolatos információk biztosítása.

Második témakör

Ezeken a felelősségeken belül, szeretnénk definiálni azokat a feladatokat, melyeket az ápolók ideális esetben végeznének. Véleménye szerint az előbbiekben definiált gyógyszerészeti ellátási felelősségek között,

• Mely specifikus feladatokat kellene az ápolóknak végeznie szerepkörükön belül a multidiszciplináris gyógyszerészeti ellátásban?

• A következő feladatokat számba véve mit változtatna meg, távolítana el vagy adna hozzá?

- Állapotváltozások észlelése, szükségletfelmérés.
- Betegfelvétel
- Multidiszciplináris kommunikáció (beleértve a jelentést, riasztást és megbeszélést)
- Kommunikáció a beteggel.

- Beavatkozás vészhelyzetben.
- Állapotkövetés.
- Az öngondoskodás támogatása.
- ’Függő’ gyógyszerfelírás.
- ’Független’ gyógyszerfelírás.
- Gyógyszerelési hibák és biztonsági problémák jelentése.

Harmadik témakör

A magas szintű multidiszciplináris gyógyszerészeti ellátás megegyezést igényel az ápolók, gyógyszerészek és orvosok közötti interakciókat illetően. Miképp írná le az ideális együttműködést és kommunikációt az ápolók és a többi egészségügyi dolgozó között, ha a magas szintű gyógyszerészeti ellátás a cél? Véleménye szerint a gyógyszerészeti ellátás keretein belül,

• Mit gondol, miből kellene állnia az együttműködésnek és kommunikációnak az ápolók és a többi egészségügyi dolgozó között?

•A következő interakciókat tekintve mit változtatna meg, távoltana el vagy adna hozzá?

- Az ápolók jelentik a megfigyeléseiket az orvosoknak és gyógyszerészeknek.
- Az orvosok információkat és instrukciókat adnak az ápolóknak.
- A gyógyszerészek tanácsokat adnak az ápolóknak.

Negyedik témakör

Megbeszéltük az ápolók felelősségeit, feladatait és szakmaközi interakcióit a multidiszciplináris gyógyszerészeti ellátásban. Ön szerint milyen előfeltételek lennének szükségesek ahhoz, hogy a felvázolt ideális helyzetet átültethessük a valóságba? Kérem, hogy vegye számításba az összes felelősséget, feladatot és multidiszciplináris interakciót.

1. Mik az ápolók szerepének erősségei a multidiszciplináris gyógyszerészeti ellátásban? Mi az, ami már most is jól megy? Mi az, amit megtartana?

2. Mik az ápolók szerepének gyengeségei a multidiszciplináris gyógyszerészeti ellátásban? Mi az, ami jelenleg nem megy jól? Mit kellene megváltoztatni az ápolók szerepkörével kapcsolatban, ha a jó minőségű gyógyszerészeti ellátás a cél?

3. Ha be szeretnénk vezetni a gyakorlatba az ápolók szerepének ideális modelljét, mik a lehetőségek? Milyen tényezők könnyítik meg a gyakorlatba való átvitelt? Milyen kedvező körülmények állnak fenn?

4. Ha be szeretnénk vezetni a gyakorlatba az ápolók szerepének ideális modelljét, mik a fenyegetések? Mi nehezíti meg a gyakorlatba való átvitelt? Milyen kedvezőtlen körülmények állnak fenn?

Interview guide

Italy

Guida all'intervista per lo studio "Un'analisi SWOT sul ruolo degli infermieri nell'assistenza farmaceutica multidisciplinare in Europa: uno studio qualitativo"

Istruzioni basate sulle discussioni durante il programma di studio intensivo, Anversa, 2018:
• Deve essere un breve questionario (7 domande (dati demografici e caratteristiche del lavoro) completato prima dell'intervista.
• Il modello sviluppato in precedenza non verrà mostrato agli informatori.
• Agli informatori verrà innanzitutto chiesto di riflettere in modo più aperto sulle responsabilità, compiti e interazioni dell'infermiere. Successivamente, chiederemo loro di riflettere sulle cose a cui avevamo pensato prima, utilizzando i contenuti specifici del modello relativo a quella domanda.
• L'intervistatore deve dichiarare chiaramente le definizioni dei concetti utilizzati e deve guidare l'informatore nella comprensione delle domande e nel rispondere ad esse nell'ambito dello scopo.
• Nessun documento può essere inviato in anticipo agli informatori.
• Tutti gli intervistatori dovrebbero essere trasparenti sulla selezione di esperti / casi critici, applicando la tecnica appresa nel programma di studio intensivo.
• Tutti i dati devono essere archiviati in modo sicuro fino a quando il coordinatore del progetto non dichiara esplicitamente che i dati possono essere rimossi.
• Eventuali problemi durante la raccolta o l'analisi dei dati devono essere segnalati al Coordinatore del progetto.
• Ti consigliamo di annotare le responsabilità, i compiti e le interazioni riportate dagli informatori in modo strutturato durante l'intervista, poiché nell'argomento 2 rifletti su tutte le responsabilità riportate dagli informatori nell'argomento 1, e nell'argomento 4 rifletti su tutte le responsabilità, i compiti e le interazioni.

Introduzione
• Dare il benvenuto al partecipante: “Ciao, benvenuto. Grazie per essere qui oggi e per esserci disposto a partecipare a questa intervista. "
• Descrivere lo studio: “Questa intervista fa parte di un'intervista europea su larga scala studio in 14 paesi sul ruolo degli infermieri nella cura farmaceutica multidisciplinare. Saranno intervistati infermieri, farmacisti e medici di diversi contesti sanitari. Il nostro obiettivo è sviluppare un modello generale per il ruolo degli infermieri nell'assistenza farmaceutica multidisciplinare, al fine di migliorare la collaborazione multidisciplinare.
Vogliamo che tu pensi a quale sarebbe il ruolo degli infermieri nell'assistenza farmaceutica multidisciplinare in una situazione ideale. Quale sarebbe l'ideale per la cura farmaceutica? Successivamente ti chiederemo di riflettere sul contesto della pratica clinica e sui prerequisiti per implementare questo ruolo. Lo faremo in modo strutturato chiedendo i punti di forza e di debolezza del ruolo degli infermieri oggi e le opportunità per il futuro. Ci concentreremo sul ruolo degli infermieri, non sul ruolo di altri professionisti sanitari. Non ci sono risposte giuste o sbagliate alle domande. È importante che tu dia la tua opinione personale sull'argomento. "
• "Posso chiederle, se possibile, di spegnere il cellulare."
• Spiegare il corso / procedura dell'intervista: “Questa intervista sarà registrata. Dopo l'intervista trascriverò l'intervista e analizzerò i dati senza il tuo nome o altri nomi che potresti aver menzionato durante l'intervista. Ora inizierò la registrazione audio. Va bene per te?"
• Chiedendo il consenso informato, dai al partecipante il tempo di leggere l'intero documento. "Posso chiederti di firmare un modulo, che si chiama" consenso informato "? Ciò significa che sei stato informato dello studio, che hai avuto abbastanza tempo per porre domande, che parteciperai volontariamente e che sai che puoi interrompere la tua partecipazione in qualsiasi momento senza fornire alcuna motivazione. Dopo aver dato il consenso informato firmando il modulo, e

completando un breve questionario con 7 domande sulle caratteristiche personali e lavorative, inizierò il colloquio, non ti chiamerò per nome durante il colloquio, che richiederà 45-60 min del tuo tempo. Puoi interrompere / mettere in pausa l'intervista se lo desideri. "

Argomento 1

Quando parliamo di cure farmaceutiche, vogliamo che tu pensi al contributo degli operatori sanitari alla cura delle persone al fine di ottimizzare l'uso dei farmaci e migliorare i risultati di salute.
Secondo lei, guardando al ruolo ideale degli infermieri nell'assistenza farmaceutica multidisciplinare,
• Quali responsabilità farebbero parte del ruolo ideale di un infermiere? Cosa implicano queste responsabilità?
• Quando si considerano le 4 responsabilità successive, cosa si desidera modificare, aggiungere o rimuovere?

Responsabilità:
- monitoraggio e follow-up degli effetti avversi / terapeutici,
- monitoraggio e follow-up dell'aderenza ai farmaci,
- processo decisionale sull'uso dei medicinali, inclusa la prescrizione di medicinali, esclusi preparazione / amministrazione e
- fornire educazione al paziente e informazioni sui medicinali.

Argomento 2

All'interno di queste responsabilità, vogliamo anche definire i compiti che gli infermieri dovrebbero idealmente eseguire.

Secondo lei, nell'ambito delle responsabilità precedentemente definite dell'assistenza farmaceutica,
• Quali compiti specifici dovrebbero svolgere gli infermieri nel loro ruolo nell'assistenza farmaceutica multidisciplinare?
• Quando si considerano le attività successive, cosa ti piace modificare, aggiungere o rimuovere?

Compiti:
- Rilevamento di cambiamenti clinici, problemi sanitari o valutazione delle esigenze dei pazienti
- Registrazione
- Comunicazione multidisciplinare (inclusi reportistica, allerta e discussione) - Comunicazione con il paziente
- Intervento in caso di emergenza
- Azione supplementare
- Supporto per l'auto-cura
- Prescrizione infermieristica "dipendente"
- Prescrizione infermieristica "indipendente"
- Segnalazione di errori relativi ai farmaci e problemi di sicurezza

• Argomento 3

L'assistenza farmaceutica multidisciplinare di alta qualità richiede accordi sull'interazione tra infermieri, medici e farmacisti. Come possiamo descrivere al meglio la collaborazione e la comunicazione ideale tra infermieri e altri professionisti sanitari, mirando a un'assistenza farmaceutica di alta qualità?
Secondo lei, nell'ambito della cura farmaceutica,
• In cosa pensi che dovrebbe consistere la collaborazione e la comunicazione tra infermieri e altri fornitori di assistenza sanitaria nell'assistenza farmaceutica multidisciplinare?
• Quando si considerano le prossime interazioni, cosa si desidera modificare, aggiungere o rimuovere?

Interazioni:
- Infermieri che riferiscono osservazioni a medici e farmacisti
- Medici che forniscono informazioni e istruzioni agli infermieri
- Farmacisti che danno consigli agli infermieri

Argomento 4

Abbiamo ora discusso sulle responsabilità, i compiti e le interazioni multidisciplinari degli infermieri nella cura farmaceutica multidisciplinare. Vorremmo che riflettessi ora sui prerequisiti per realizzare questo modello ideale.
Si prega di considerare tutte le responsabilità, i compiti e le interazioni multidisciplinari.

1. Quali sono i punti di forza del ruolo degli infermieri nell'assistenza farmaceutica multidisciplinare? Cosa sta già andando bene? Cosa vorresti conservare?
2. Quali sono i punti deboli del ruolo degli infermieri nell'assistenza farmaceutica multidisciplinare? Cosa non sta andando bene in questo momento? Cosa dovrebbe essere cambiato nel ruolo degli infermieri nel mirare a cure farmaceutiche di alta qualità.
3. Se volessimo mettere in pratica il modello ideale del ruolo degli infermieri nell'assistenza farmaceutica multidisciplinare, quali sono le opportunità? Cosa rende più facile realizzare l'implementazione? Quali sono le circostanze favorevoli?
4. Se volessimo mettere in pratica il modello ideale del ruolo degli infermieri nell'assistenza farmaceutica multidisciplinare, quali sono le minacce? Cosa rende più difficile realizzare l'implementazione? Quali sono le circostanze sfavorevoli?

Interview guide

North Macedonia

Водич за интервју за студијата “SWOT анализа на улогата на медицинските сестри во мултидисциплинарната фармацевтска нега во Европа: квалитативна студија“

Инструкции базирани на дискусиите за време на интензивната програма на студијата, Антверп, 2018:

- Краток прашалник (7 прашања (демографски и работни карактеристики)) мора да биде пополнет пред интервјуто.
- Моделот кој што беше развиен однапред, нема да биде покажан на информаторите.
- Од информаторите ќе биде побарано да се изразат поотворено за обврските, задачите и интеракциите на медицинските сестри. После тоа, ќе бидат прашани за работите што беа смислени однапред, употебувајќи го моделот што кореспондира со наведеното прашање.
- Интервјуто мора јасно да ги наведе дефинициите на концептите што се употребени, и мора да го насочи информаторот кон разбирање на прашањето и нивниот одговор да одговара на рамките на прашањето.
- Никакви документи нема да им бидат дадени на информаторите пред интервјуто.
- Сите интервјуери треба да бидат транспарентни за нивната селекција на експертски/критични случаи, и да ги аплицираат техниките кои ги научија во интензивната студија.
- Сите податоци мора да бидат соодветно складирани, се додека координаторот на проектот на писмено не наведе дека податоците можат да бидат отстранети.
- Сите проблеми поврзани со собирањето на податоци или анализа на податоците мора да бидат пријавени кај координаторот на проектот.

- Ве советуваме на структуриран начин да ги запишувате обврските, задачите и интеракциите кои што ќе бидат спомнати од информаторите за време на интервјуто, бидејќи во тема 2 ќе треба да се спомнат обврските наведени во тема 1, и во тема 4 - сите обврски, задачи и интеракции треба да се повторат.

Вовед:

- Дочек на учесниците: „Здраво, добредојдовте. Ви благодариме што сте тука денеска и што се одлучивте да учествувате на интервјуто“.
- Опис на студијата: „Ова интервју е дел од една голема Европска студија која што се спроведува во 14 земји, за улогата на медицинската сестра во мултидисциплинарната фармацевтска нега. Ќе бидат интервјуирани медицински сестри, фармацевти и лекари од различни специјалности. Нашата цел е да развиеме еден генерален модел за улогата на медицинските сестри во мултидисциплинарната фармацевтска нега, за да можеме да ја подобриме мултидисциплинарната колаборација. Сакаме да размислите и да ни кажете што за вас претставува улогата на медицинските сестри во мултидисциплинарната фармацевтска нега во една идеална ситуација. Што би било идеално во фармацевтската нега? После тоа ќе бидете прашани во контекстот на улогата на медицинската сестра во клиничката пракса и за предусловите за имплементација на оваа улога. Ќе го направиме ова на еден структуриран начин, истовремено прашувајќи за предностите и слабостите на улогата на медицинските сестри денеска, и за можностите за нивен напредок во иднина. Ќе се фокусираме на улогата на медицинските сестри, а не на улогата на другиот медицински персонал. Нема добри или лоши одговори на прашањата. Единствено важно е да го дадете вашето лично мислење на темата.“.

- „Би ве замолил да ги исклучите вашите мобилни телефони“
- Објаснување за текот/процедурата на интервјуто: „Ова интервју ќе се снима на аудиозапис. После интервјуто, вашето излагање ќе биде конвертирано во текст и ќе биде анонимно анализирано, без да биде вклучено вашето име или имињата кои што сте ги спомнале за време на интервјуто. Дали е ова прифатливо за вас?“
- Барање на писмена согласност: Дајте му време на учесниците да го прочитаат целиот документ. „Би ве замолил да го потпишете овој формулар, насловен како „писмена согласност“? Ова значи дека вие сте информирани за студијата, дека имавте доволно време да поставите прашања, дека доброволно учествувате, и дека можете да го прекинете вашето учество во било кое време од интервјуто без да треба да наведете причина за тоа. По давањето на писмена согласност со потпишување на формуларот, и комплетирањето на краткиот прашалник од 7 прашања за демографските и работните карактеристики, ќе го започнеме интервјуто. Вашето име нема да биде спомнато за време на интервјуто, што ќе трае негде околѕ 45-60 минути. Вие ќе можете да го запрете/паузирате интервјуто во било кое време.

Тема 1

Кога зборуваме за фармацевтска нега, сакаме да размислите за придонесот на здравствените работници во негата на пациентите, со цел да се оптимизира употребата на лекарства и да се подобри здравствениот исход на крајот на лечењето на пациентот.

По ваше мислење, гледајќи на улогата на медицинските сестри во мултидисциплинарната фармацевтска нега,

- Кои обврски треба да бидат дел од идеалната улога на медицинската сестра? Што треба овие обврски да значат во контекст на негата?

- Кога би ги имале во предвид следните 4 обврски, што би сакале да промените, додадете или одземете од листата?

  - Мониторирање и следење на несакани / терапевтски ефекти,
  - Мониторирање и следење на лековите и придржување до терапијата
  - Носење на одлуки за употребата на медикаменти, вклучувајќи препишување на лекови, исклучувајќи спремање и администрација на лекови
  - Информирање и едукација на пациентот за медикаментите во неговата терапија

Тема 2

Во однос на обврските, исто така би сакале да ги дефинираме задачите што една медицинска сестра би требало да ги извршува при едни идеални услови.

По ваше мислење, со претходно дефинираните обврски од фармацевтската нега:

- Кои специфични задачи би требало една медицинска сестрада ги извршува во нејзината улога во мултидисциплинарната фармацевтска нега?

- Кога би ги имале во предвид следните работни задачи, што би сакале да промените, додадете или одземете од листата?
  - Откривање на клинички промени, здравствени проблеми или проценка на потребите на пациентот
  - Регистрација
  - Мултидисциплинарна комуникација (вклучувајќи известување, алармирање и дискусија)
  - Комуникација со пациент
  - Интервенција за време на итен случај
  - Следење
  - Поддршка при самостојното земање на лекови од страна на пациентот
  - „Зависно“(под надзор) препишување на лекови од страна на медицинската сестра
  - Независно препишување на лекови од страна на медицинската сестра
  - Известување за грешки во лековите и проблеми со сигурноста

Тема 3

Висококвалитетната мултидисциплинарна фармацевтска нега бара утврдена интеракција помеѓу медицинските сестри, докторите и фармацевтите. Како најдобро би можел да ја опишеш идеалната колаборација помеѓу медицинските сестри и останатиот медицински персонал, со цел да се постигне високо квалитетна фармацевтска нега?

По ваше мислење, во доменот на фармацевтската нега:

- Во што би требало да се состои колаборацијата и комуникацијата меѓу медицинските сестри и останатиот медицински персонал во однос на мултидисциплинарната фармацевтска нега?
- Кога би ги имале во предвид следните интеракции, што би сакале да промените, додадете или одземете од листата?
    - Медицинските сестри да ги известуваат лекарите и фармацевтите за нивните обсервации
    - Лекарите да даваат информации и инструкции на медицинските сестри
    - Фармацевтите да даваат совети на медицинските сестри

Тема 4

Досега дискутиравме за обврските, задачите и интеракциите на медицинските сестри во однос на мултидисциплинарната фармацевтска нега. Сега би сакале да ве прашаме за тоа кои би биле предусловите за да овој идеален модел на нега кој што го дискутиравме, да биде пресликан во реалноста.

Ве молиме разгледајте ги сите обврски, задачи и мултидисциплинарни интеракции:

1. Кои се предностите на улогата на медицинските сестри во мултидисциплинарната фармацевтска нега? Што досега било добро во однос на ова? Што би сакале да задржите?

2. Кои се слабостите на улогата на медицинските сестри во мултидисциплинарната фармацевтска нега? Што досега не оди добро во однос на ова? Што би требало да се промени во улогата на медициските сестри со цел да се постигне висококвалитетна фармацевтска нега.

3. Доколку би се одлучиле овој идеален модел улогата на медицинската сестра во мултидисциплинарната фармацевтска нега да го применеме во пракса, кои би биле можностите за тоа? Што би ја олеснило реализацијата на овој модел? Кои би биле олеснителните околности?

4. Доколку би се одлучиле овој идеален модел улогата на медицинската сестра во мултидисциплинарната фармацевтска нега да го применеме во пракса, кои би биле ризиците? Што би ја отежнало реализацијата на овој модел? Кои би биле околностите кои би го отежнале целиот овој процес?

Interview guide

Norway

Intervjuguide for studien ”En SWOT analyse om sykepleieres rolle i multiprofesjonell farmasøytisk omsorg i Europa: en kvalitativt studie»

Instruksjoner basert på diskusjonen i Antwerpen:

- Et kort spørreskjema (7 spørsmål om demografi og jobbeskrivelser) må fylles ut før intervjuet setter i gang.
- Modellen som ble lagd på forhånd vil ikke bli vist til informantene.
- Informantene vil først bli spurt om å reflektere mer åpent over sykepleiernes ansvar, oppgaver og samspill. Etterpå vil vi be de reflektere over det spesielle innholdet knyttet til modellen.
- Intervjueren må klargjøre begrepene som blir brukt og må guide informantene til å forstå spørsmålene slik at tematikken blir ivaretatt.
- Ingen dokumenter vil bli sendt til informantene på forhånd.
- Alle intervjuere må være transparente med tanke på utvelgelse av eksperter som tilsvarer opplæringen på det intensive studie program.
- Alle data skal oppbevares på en sikker måte frem til prosjekt- koordinatoren uttrykkelig gir skriftlig informasjon om at de kan slettes.
- Ethvert problem/utfordring i prosessen med datainnsamling eller dataanalyse skal videreformidles til koordinatoren.
- Vi anbefaler dere å notere ansvarsområder, oppgaver og samspill beskrevet av informantene på en strukturert måte i løpet av intervjuet, som i tema 2 hvor du spør om alle ansvarsområder rapportert av informanten i tema 1, og i tema 4 hvor du spør om alle ansvarsområder, oppgaver og samspill.

Introduksjon:

Velkommen til deltakerne. «Hei, velkommen. Tusen takk for at du er her i dag, og er villig til å delta i dette intervjuet.»

Beskrivelse av studien: «Dette intervjuet er en del av en større europeisk intervju studie som foregår i 14 land, som handler om sykepleierens rolle i tverrfaglig farmasøytisk omsorg (legemiddelhåndtering). Sykepleiere, leger og farmasøyter i ulike helsetjenester vil bli intervjuet.
Hensikten med studien er å utvikle en generell modell for sykepleiers rolle i tverrfaglig farmasøytiske omsorg (legemiddelhåndtering), for å kunne bedre det tverrfaglige samarbeidet. Vi ønsker at du tenker over hva sykepleiers rolle i farmasøytisk omsorg bør være i en ideal situasjon. Hva vil være ideelt for farmasøytisk omsorg? Etterpå vi vil be deg om å reflektere over sammenhengen mellom klinisk praksis og forutsetningene for å gjennomføre denne rollen. Vi vil gjøre dette på en strukturert måte ved å spørre etter styrker og svakheter for sykepleiers rolle i dag, og om muligheter og trusler for fremtiden. Vi vil fokusere på sykepleiers rolle, og ikke på andre helsepersonells rolle. Det er ingen riktige eller gale svar på spørsmålene. Det er viktig at du gir oss din personlige mening på emnet.

«Kan jeg spørre deg, hvis mulig, til å skru av mobiltelefonen din.»

Forklaring av fremgangsmåten/ prosedyren av intervjuet: «Dette intervjuet vil bli tatt opp.» Etter intervjuet vil jeg skrive ut intervjuet og analysere dataene uten ditt navn eller andre navn som du kanskje har nevnt under intervjuet. Jeg vil nå starte lydbåndet. Er det greit for deg?»

Be om informert samtykke, gi deltakerne tid til å lese hele dokumentet. «Kan jeg be deg om å signere et skjema, det kalles «informert samtykke»? Dette betyr at du har blitt informert om studien, at du hadde nok tid å stille spørsmål, at du vil delta frivillig og at du vet at du kan trekke din deltakelse når som helst uten å oppgi noen grunn. Etter du har signert samtykke dokumentet og fullført et kort spørreskjema bestående av 7 spørsmål om personopplysninger og jobb beskrivelser, vil jeg starte intervjuet. Jeg vil ikke bruke navnet ditt under intervjuet, og intervjuet vil ta ca 45 til 60 minutter av tiden din. Du kan stoppe, ta pause under intervjuet hvis du ønsker det.

Emne 1:

Når vi snakker om farmasøytisk omsorg (legemiddel omsorg), ønsker jeg at du tenker på helsepersonells bidrag til omsorg for den enkelte for å optimalisere bruken av medisiner og bedre pasientens helse.

Hva er din oppfattelse av sykepleierens ideelle rolle i et tverrfaglig farmasøytisk arbeid?

- Hvilke oppgaver (ansvarsområder) vil være en del av en ideel sykepleierrolle? Hva innebærer dette ansvaret?
- Når du vurderer de neste 4 ansvarsområder, hva vil du endre, legge til eller fjerne?

  - overvåking og oppfølging av uønskede / terapeutiske effekter,
  - overvåkning og oppfølging av overholdelse av medisineringen (riktig medisinbruk)
  - beslutningsprosesser om bruk av medisin, inkludert forskrivning av medisiner, men unntatt forberedelse / administrasjon, og
  - pasientopplæring og informasjon om medisiner.

Emne 2:

Med tanke på de fire ansvarsområder, ønsker jeg at du skal definere de oppgaver som sykepleieren ideelt sett bør utføre.

Etter din mening, med den førnevnte definisjon av farmasøytisk omsorg (legemiddel omsorg?) in mente;

- Hvilke spesifikke oppgaver bør sykepleiere utføre i tverrfaglig farmasøytisk omsorg (legemiddel omsorg)?
- Når du vurderer følgende oppgaver, er det da noe du vil du endre, tilføye eller ta bort?

  Oppgaver:

  - Oppdage kliniske endringer, helseproblemer eller vurdering av pasientens behov

- Registrering (dokumentasjon)
- Tverrfaglig kommunikasjon (inkludert rapportering, varsling og diskusjon)
- Pasientkommunikasjon
- Intervensjoner i akuttsituasjoner
- Oppfølgning
- Selfcare support
- 'Avhengige' sykepleier forskrivning
- 'Uavhengig' sykepleier forskrivning
- Rapportering av medisineringsfeil og sikkerhets utfordringer

Emne 3

Høyt kvalitet i tverrfaglig farmasøytisk omsorg krever enighet om samspillet mellom sykepleiere, leger og farmasøyter. Hvordan kan vi best beskrive det ideelle samarbeidet og kommunikasjonen mellom sykepleiere og andre helsepersonell, med sikte på høyt kvalitet av farmasøytisk omsorg?

Etter din mening, innenfor domenet av farmasøytisk omsorg

- Hva tenker du at samarbeid og kommunikasjon mellom sykepleiere og annet helsepersonell i tverrfaglig farmasøytisk omsorg bør bestå av?
- Når du vurderer de neste punktene om samhandling, hva vil du endre, legge til eller fjerne?

Samhanding:
- Sykepleiere rapporterer observasjoner til leger og farmasøyter
- Leger gir informasjon og instruksjoner til sykepleiere
- Farmasøyter gir råd til sykepleiere

Emne 4

Vi har nå snakket om sykepleiers ansvar, oppgaver og samhandling i tverrfaglig farmasøytisk omsorg. Vi ønsker nå at du skal reflektere over forutsetningene for å bringe denne ideelle modellen til virkelighet.

Vær vennlig å vurder alle ansvarsområder, oppgaver og tverrfaglig samhandling.

1. Hva er styrken i sykepleierens rolle i tverrfaglig farmasøytisk omsorg? Hva fungerer allerede? Hva vil du beholde?
2. Hva er svakheter i sykepleierens rolle i tverrfaglig farmasøytisk omsorg? Hva fungerer ikke? Hva bør endres i sykepleierens rolle med sikte på høy kvalitet i farmasøytisk omsorg?
3. Hvis vi ønsker å sette den ideelle modellen i sykepleiers rolle i tverrfaglig farmasøytisk omsorg ut i praksis, hva er mulighetene? Hva kan gjøre det enklere å realisere implementeringen? Hvilke forhold kan fremme implementeringen?

4. Hvis vi ønsker å sette den ideelle modellen til sykepleiers rolle i tverrfaglig farmasøytisk omsorg ut i praksis, hva er barrierene? Hva kan gjøre det vanskeligere å realisere implementeringen? Hvilke forhold kan hemme implementeringen?

Interview guide

Portugal

Interview guide DeMoPhaC - Portugal

Guião de Entrevista: Análise SWOT sobre o papel dos enfermeiros no cuidado farmacoterapêutico interdisciplinar em 14 países europeus: um estudo qualitativo

Introdução

• Acolher o participante: **"Olá, seja bem-vindo. Obrigado por estar aqui hoje e estar disposto a fazer parte desta investigação, participando nesta entrevista."**

• Descrever o estudo: **O meu nome é XXX, sou investigador na Escola Superior de Enfermagem de Coimbra.**

Esta entrevista faz parte da colheita de dados de um projeto europeu em larga escala sobre o papel dos enfermeiros no cuidado farmacoterapêutico interdisciplinar. Enfermeiros, médicos e farmacêuticos de diferentes instituições e contextos de saúde serão entrevistados.

O objetivo da investigação é desenvolver um modelo geral orientador para o papel do enfermeiro no cuidado farmacoterapêutico interdisciplinar, a fim de melhorar a colaboração e os cuidados prestados.

Só uma breve contextualização: Entendemos sucintamente por cuidado farmacoterapêutico - tudo o que envolve: a avaliação da necessidade do doente, a prescrição, o armazenamento, a preparação e a administração do medicamento, a avaliação dos efeitos terapêuticos e adversos, a educação/formação do doente para a auto-medicação e o follow-up e verificação da adesão ao tratamento.

O papel dos enfermeiros no cuidado farmacoterapêutico interdisciplinar não é transparente e varia entre os vários países europeus. Da mesma forma, na educação do enfermeiro, falta uma descrição clara dos resultados específicos da aprendizagem, e os conteúdos formativos sobre os cuidados farmacoterapêuticos variam muito de país para país. Além disso, a correspondência com as necessidades do mercado de trabalho e da sociedade revela-se insuficiente. A falta de transparência e reconhecimento, juntamente com a variação entre os países, na prática e educação de enfermagem tem um grande impacto. Dificulta a colaboração em diferentes níveis: colaboração interdisciplinar na prática clínica; colaboração transnacional em investigação, educação e inovação na Europa; mobilidade laboral dos enfermeiros. Como resultado, a segurança do doente é ameaçada, o desenvolvimento e a inovação não são tão ágeis e os orçamentos de saúde não são usados da forma mais eficiente.

Numa análise preliminar dos dados do projeto EUPRON (de 3300 enfermeiros europeus, médicos e farmacêuticos), que deu origem ao DeMoPhaC, estes mostram que quase todos os participantes estão convencidos do impacto positivo sobre a qualidade dos cuidados de um maior envolvimento dos

enfermeiros no cuidado farmacoterapêutico. Além desta percepção, a comunicação interprofissional revelou um resultado de 5,2/10, uma pontuação alarmante dada a ligação entre a segurança do doente e comunicação interprofissional.

(...)

Queremos que reflita sobre o papel ideal dos enfermeiros no cuidado farmacoterapêutico interdisciplinar. O que seria ideal nos cuidados farmacoterapêuticos? Em seguida, pedir-lhe-emos que reflita sobre o contexto da prática clínica e os pré-requisitos para implementar esse papel. Procurando estruturar e solicitando na sua opinião: as forças e fraquezas do papel dos enfermeiros hoje e, sobre as oportunidades e ameaças no futuro. Vamos concentrar-nos no papel dos enfermeiros, não no papel de outros profissionais de saúde.

Não existem respostas corretas ou erradas sobre as questões que colocarei. É importante que descreva a sua opinião pessoal sobre o tema."

• Explicando o curso /procedimento da entrevista: **"Esta entrevista será gravada em áudio. Permite-o?**

- **Sim.**

Após a entrevista esta será transcrita e analisada sem referência ao seu nome ou outros nomes que possa ter mencionado durante a entrevista. Posso começar a gravação?"

• Pedir consentimento informado, fornecer ao participante tempo para ler o documento na totalidade.

"Posso pedir que assine um formulário de 'consentimento informado'? Significando que foi informado sobre o estudo, que teve tempo suficiente para fazer perguntas, que vai participar voluntariamente e que que pode suspender sua participação em qualquer momento sem dar qualquer justificação.

(…)

Podemos interromper a entrevista sempre que considerar oportuno, solicite uma paragem se o pretender."

<u>TÓPICO 1</u>

Quando falamos em cuidado farmacoterapêutico, queremos que reflita sobre a contribuição dos profissionais de saúde para a optimização da utilização de medicamentos e maximização de ganhos em saúde.

Na sua opinião, em relação ao papel ideal do enfermeiro no cuidado farmacoterapêutico:

- Quais as responsabilidades que fariam parte desse papel? O que implicariam essas responsabilidades?

Accepted by Ethics Committee EAHSW on 30 Oct 2018

Considerando as quatro responsabilidades que se seguem, gostaria de alterar, acrescentar ou remover alguma delas do papel ideal do enfermeiro no cuidado farmacoterapêutico?

Responsabilidades:

- Monitorizar e acompanhar efeitos adversos e terapêuticos;
- Monitorizar e acompanhar adesão terapêutica;
- Tomada de decisão no uso de medicação, incluindo prescrição medicamentosa, para além da preparação e administração;
- Realizar ensinos e providenciar informação acerca dos medicamentos.

Responsabilidades:

- Monitorizar e acompanhar efeitos adversos e terapêuticos;
- Monitorizar e acompanhar adesão terapêutica;
- Tomada de decisão no uso de medicação, incluindo prescrição medicamentosa, para além da preparação e administração;
- Realizar ensinos e providenciar informação acerca dos medicamentos.

TÓPICO 2

Dentro destas responsabilidades, temos ainda como objetivo definir tarefas que os enfermeiros iriam idealmente realizar.

Na sua opinião, com a definição prévia das responsabilidades no cuidado Farmacoterapêutico:

2.1 – Quais as tarefas específicas que os enfermeiros deveriam realizar ao desempenhar a sua função no Cuidado Farmacoterapêutico?

2.2 – Considerando as seguintes tarefas, o que gostaria de mudar, adicionar ou eliminar?

Tarefas:

- Detetar mudanças clínicas, problemas de saúde, avaliar as necessidades do utente;
- Registos;
- Comunicação interprofissional (incluindo relatos, alertas e discussão);
- Comunicação com o utente;
- Intervenções no caso de emergência;
- Acompanhamento;

- Apoio no auto-cuidado;

- Prescrições de enfermagem dependentes;

- Prescrições de enfermagem independentes;

- Relato de erros medicamentos e problemas de segurança.

TÓPICO 3

Cuidado interprofissional farmacoterapêutico de qualidade requer a concordância de interações entre enfermeiros, médicos e farmacêuticos. Como é que podemos descrever a comunicação e colaboração ideal entre enfermeiros e outros profissionais de saúde, tendo como objetivo o cuidado farmacoterapêutico de qualidade?

Na sua opinião, dentro do domínio de Cuidado Farmacoterapêutico,

3.1 – Em que consiste a colaboração e comunicação entre enfermeiros e outros profissionais de saúde no cuidado interprofissional farmacoterapêutico?

3.2 – Considerando as próximas interações, o que gostaria de mudar, adicionar ou eliminar?

Interações:

- Relato de observações, pelos enfermeiros, a médicos e farmacêuticos;

- Providenciar informações e instruções, pelos médicos aos enfermeiros.

- Conselhos dados pelos farmacêuticos aos enfermeiros.

TÓPICO 4

Até agora discutimos sobre as responsabilidades, tarefas e interações interprofissionais dos enfermeiros no cuidado farmacoterapêutico interprofissional.

Gostaríamos agora de refletir nos pré-requisitos necessários para aplicar este modelo no contexto real.

Considerando as responsabilidades, tarefas e interações interprofissionais:

1 - Quais os pontos fortes no papel do enfermeiro no cuidado farmacoterapêutico interprofissional? O que é que já está a correr bem? O que gostaria de manter?

1 Quais os pontos fracos no papel do enfermeiro no cuidado farmacoterapêutico interprofissional? O que é que não está a correr bem no momento? O que deveria ser mudado no papel do enfermeiro com o objetivo de um cuidado farmacoterapêutico de qualidade?

2 Se se pusesse em prática o modelo do papel do enfermeiro no cuidado farmacoterapêutico interprofissional, quais seriam as oportunidades? O que facilitaria a sua implementação? Quais seriam as circunstâncias favoráveis?

3 Se se pusesse em prática o modelo do papel do enfermeiro no Cuidado Farmacoterapêutico interprofissional, quais seriam as ameaças? O que dificultaria a sua implementação? Quais seriam as circunstâncias desfavoráveis?

Conclusão:

"Obrigado, novamente, por se ter disponibilizado a participar nesta investigação."

Interview guide

Slovakia

Sprievodca rozhovorom o štúdii "SWOT analýza úlohy sestier v multidisciplinárnej farmaceutickej starostlivosti v Európe: kvalitatívna štúdia"

Pokyny založené na diskusiách počas intenzívneho študijného programu, Antverpy, 2018:

- Pred rozhovorom je potrebné dať vyplniť krátky dotazník (7 otázok -demografické údaje a charakteristiky pracovných miest).
- Model, ktorý bol vyvinutý vopred, sa informátorom nezobrazí.
- Respondenti budú najskôr požiadaní, aby sa otvoreným spôsobom vyjadrili k zodpovednosti sestier, k ich úlohe a interakcii. Následne ich požiadame, aby sa zamysleli nad vecami, o ktorých sme už predtým hovorili, s použitím konkrétneho obsahu modelu súvisiaceho s touto otázkou.
- Anketár musí jednoznačne uviesť definície použitých pojmov a musí respondenta viesť k pochopeniu otázok a odpovedať na ne v požadovanom rozsahu.
- Žiadne dokumenty nesmú byť zaslané respondentovi vopred.
- Všetci anketári by mali byť transparentní pri výbere expertných / kritických prípadov, pričom by sa mali uplatňovať techniky, ktoré sa naučili v intenzívnom študijnom programe.
- Všetky údaje by mali byť uchovávané bezpečným spôsobom, kým koordinátor projektu výslovne písomne neuvedie, že dáta môžu byť odstránené.
- Všetky problémy počas zbierania dát alebo ich analýze by sa mali oznamovať koordinátorovi projektu.
- Odporúčame Vám, aby ste počas rozhovoru štruktúrovaným spôsobom zapísali zodpovednosti, úlohy a interakcie, ktoré respondenti oznámili, pretože v téme 2 sa zamyslíte nad všetkými uvedenými zodpovednosťami respondentov v téme 1 a v téme 4 nad všetkými zodpovednosťami, úlohami a interakciami.

Predstavenie

- **<u>Privítanie účastníkov</u>**: Dobrý deň, vitajte. Ďakujeme Vám, že ste dnes prišli a boli ochotní sa zúčastniť dnešného rozhovoru.
- **<u>Popis štúdie:</u>** Tento rozhovor je časťou rozhovorov, ktoré sa konajú vo veľkom meradle v 14 krajinách Európy o úlohe sestier v multidisciplinárnej farmaceutickej starostlivosti.

Tejto štúdie sa zúčastňujú sestry, farmaceuti a lekári rôznych zdravotníckych zariadení.
Naším cieľom je vytvoriť všeobecný model úlohy sestier v multidisciplinárnej farmaceutickej starostlivosti s cieľom zlepšiť multidisciplinárnu spoluprácu.
Chceme, aby ste porozmýšľali o tom, aké úlohy sestier v multidisciplinárnej farmaceutickej starostlivosti by boli v ideálnej situácii.
Čo by bolo ideálne pre farmaceutickú starostlivosť?
Potom vás požiadame, aby ste sa zamysleli nad kontextom klinickej praxe a predpokladmi na vykonanie tejto úlohy.
Budeme to robiť cestou štruktúrovaného rozhovoru, v ktorom sa budeme pýtať na silné a slabé stránky úlohy sestier v súčasnosti a na príležitosti a hrozby do budúcnosti.
Zameriavame sa na úlohu sestier, nie na úlohu ostatných zdravotníckych pracovníkov. Na otázky nie sú správne ani nesprávne odpovede.

- Je dôležité, aby ste na túto tému vyjadrili svoj osobný názor.
- Môžem vás požiadať, ak je to možné, vypnúť mobilný telefón.
- <u>Vysvetlenie priebehu / postupu rozhovoru:</u> "Tento rozhovor bude nahrávaný. Po rozhovore spíšem záznam bez vášho mena alebo iných mien, ktoré by ste mohli spomenúť počas rozhovoru. Teraz zapnem nahrávanie. Vyhovuje Vám to? "
- Ak žiadate o informovaný súhlas, poskytnite účastníkovi čas na prečítanie celého dokumentu. "Môžem vás požiadať, aby ste podpísali formulár, ktorý sa nazýva" informovaný súhlas "? To znamená, že ste boli informovaný o štúdii, že máte dostatok času na to, aby ste sa pýtali na otázky, že sa zúčastňujete na dobrovoľnej báze a že viete, že môžete kedykoľvek ukončiť svoju účasť bez udania dôvodu. Po udelení informovaného súhlasu podpísaním formulára začnem rozhovor, nebudem vám hovoriť menom počas rozhovoru, čo bude trvať 45-60min času. Rozhovor môžete zastaviť / pozastaviť, ak budete chcieť. "

- **<u>Téma 1</u>**

Keď hovoríme o farmaceutickej starostlivosti, chceme, aby ste porozmýšľali o….:

Podľa vášho názoru pri pohľade na ideálnu úlohu sestier v multidisciplinárnej farmaceutickej starostlivosti,

- Aké zodpovednosti by boli súčasťou ideálnej úlohy sestry? Čo tieto zodpovednosti znamenajú?
- Pri zvažovaní ďalších 4 zodpovedností -ktoré by ste chceli zmeniť, pridať alebo odstrániť?

Zodpovednosti:

Responsibility 1-
monitorovanie a sledovanie vedľajších / terapeutických účinkov,

- **Responsibility 2**
monitorovanie a sledovanie dodržiavania užívania liekov,

Responsibility 3
poskytovanie vzdelávania pacientov a informácií o liekoch.

Responsibility 4
rozhodovanie o užívaní liekov, vrátane predpisovania liekov, s výnimkou prípravy / podávania liekov,

písať do riadku Description of responsibility

Responsibility 5

Responsibility 6

Responsibility 7

Ďalej uvádzať ako „Responsibility additional“

- **<u>Téma 2</u>**

V rámci týchto zodpovedností chceme tiež definovať/ určiť úlohy, ktoré by sestry v ideálnom prípade vykonávali.

Podľa Vášho názoru v rámci predtým definovaných povinností farmaceutickej starostlivosti…:

- Ktoré špecifické úlohy by mali sestry vykonávať vo svojej úlohe v multidisciplinárnej farmaceutickej starostlivosti?
- Ak zvažujete ďalšie úlohy, čo by ste chceli zmeniť, pridať alebo odstrániť?

Úlohy:
- zisťovanie klinických zmien, zdravotných problémov alebo posúdenie potrieb pacienta
- dokumentácia
- multidisciplinárna komunikácia (vrátane podávania správ, upozornení a diskusií)
- komunikácia s pacientmi
- zásah v prípade núdze
- sledovanie a následné opatrenia
- podpora vlastnej starostlivosti pacienta
- "závislá" predpisujúca sestra
- "nezávislá" predpisujúca sestra
- hlásenie chýb pri podávaní liekov a otázky bezpečnosti

Responsibility collaboration

- **<u>Téma 3</u>**

Vysokokvalitná multidisciplinárna farmaceutická starostlivosť vyžaduje dohodu o interakcii medzi sestrami, lekármi a farmaceutmi. Ako môžeme najlepšie opísať ideálnu spoluprácu a komunikáciu medzi sestrami a inými odborníkmi v oblasti zdravotnej starostlivosti zameranou na vysoko kvalitnú farmaceutickú starostlivosť?

Podľa Vášho názoru v oblasti farmaceutickej starostlivosti:

- Čo si myslíte, z čoho by mala pozostávať spolupráca a komunikácia medzi sestrami a inými poskytovateľmi zdravotnej starostlivosti v multidisciplinárnej farmaceutickej starostlivosti?
- Ak zvažujete ďalšie interakcie, čo by ste chceli zmeniť, pridať alebo odstrániť?

Interakcie:
- sestry, ktoré hlásia pozorovania lekárom a farmaceutom

- lekári, ktorí poskytujú informácie a pokyny sestrám
- farmaceuti, ktorí poskytujú rady sestrám

- **Téma 4**

Teraz sme diskutovali o zodpovednosti, úlohách a multidisciplinárnych vzťahoch sestier v multidisciplinárnej farmaceutickej starostlivosti. Chceli by sme, aby ste teraz uvažovali o predpokladoch, ako by sa tento ideálny model uviedol do reality.

Prosíme zvážte všetky povinnosti, úlohy a multidisciplinárne interakcie.

1. Aké sú **Strengths** silné stránky úlohy sestier v multidisciplinárnej farmaceutickej starostlivosti? Čo funguje dobre? Čo by ste chceli ponechať?
2. Aké sú **Weaknesses** slabé stránky sestier v multidisciplinárnej farmaceutickej starostlivosti? Čo v súčasnosti nie je dobre? Čo by sa malo zmeniť v úlohe sestry v snahe o vysokokvalitnú farmaceutickú starostlivosť.
3. **Opportunities** Ak by sme chceli preniesť ideálny model úlohy sestry v multidisciplinárnej farmaceutickej starostlivosti do praxe, aké sú možnosti? Čo uľahčuje realizáciu implementácie? Aké sú priaznivé okolnosti? = príležitosti
4. **Threats** Ak by sme chceli preniesť do praxe ideálny model úlohy sestry v multidisciplinárnej farmaceutickej starostlivosti, aké sú hrozby? Čo sťažuje realizáciu implementácie? Aké sú nepriaznivé okolnosti? = hrozby

Interview guide

Slovenia

VODIČ PO INTERVJUJU

SWOT analiza medpoklicnega sodelovanja medicinskih sester v okviru farmacevtske skrbi v 14 evropskih državah: kvalitativna raziskava

Uvodni pozdrav: Lepo pozdravljeni, hvala, ker ste pripravljeni sodelovati z nami pri raziskavi.

- **Opis študije:** Ta intervju je del široko obsežne evropske raziskave, ki poteka v 14ih državah in se osredotoča na vloge medicinskih sester v medpoklicnem sodelovanju na področju farmacevtske skrbi oziroma rokovanju z zdravili v Evropi. V raziskavo so vključeni zdravniki, farmacevti ter medicinske sestre različnih delovnih ozadij. Naš cilj je razvoj modela, ki bi pripomogel k izboljšavam in napredku multidisciplinarnega sodelovanja pri rokovanju z zdravili ter izobraževanja za medicinske sestre. Za rdečo nit intervjuja bomo uporabili prototipno verzijo tega modela v nastajanju, ki je bil razvit v sodelovanju z zastopniki vseh 14ih sodelujočih držav. Ta model je namenjen zgolj olajšani komunikaciji, v nobenem primeru ni obvezujoč in na njem lahko spreminjate karkoli bi želeli oziroma kar bi po vašem mnenju moralo biti drugače. Želimo si vašega mnenja o vlogi medicinske sestre pri rokovanjuv z zdravili v idealnem svetu, kaj bi bilo idealno za pacientovo preskrbo z zdravili? Nato Vas bomo prosili, da se osredotočite na realnost klinične prakse ter kaj bi se moralo zgoditi oziroma spremeniti, da bi vaše predloge lahko uvedli v prakso. Intervju je strukturiran tako, da se osredotoča na prednosti, pomanjkljivosti, nevarnosti in priložnosti, ki se ponujajo v prihodnosti. Študija bo osredotočena samo na vlogo medicinske sestre. Tukaj ni pravilnih in napačnih odgovorov, najbolj nam je pomembno, da slišimo Vaše osebno mnenje.
- Če je možno, bi Vas prosil/a, da utišate svoj mobilni telefon
- Intervju bo glasovno posnet, kasneje bo pretipkan in podatki bodo obdelani, pri čemer bodo vsa imena, vključno z vašim izvzeta. Se strinjate, da sedaj pričnem snemati pogovor?
- **Informirani pristanek:** prosil/a bi vas, da podpišete pisno soglasje o prostovoljnem sodelovanju, kar pomeni, da ste bili o študiji informirani, ste imeli možnost za postavljanje morebitnih dodatnih vprašanj in, da se zavedate, da lahko kadarkoli prekinete sodelovanje pri raziskavi, brez navedbe razlogov za to. Med intervjujem vas ne bom nazival/a z imenom, intervju pa bo trajal okoli 45-60 minut. Med intervjujem lahko kadarkoli zaprosite za pavzo.

<u>Tema 1</u>

(Želimo razviti model za vlogo medicinske sestre v medpoklicnem sodelovanju pri rokovanju z zdravili, zato je za nas pomembno, da izvemo kakšna naj bi bila ta vloga v idealnih pogojih, kakšni bi naj bili idealni pogoji za najkvalitetnejšo rokovanje z zdravili? Pri tem smo osredotočeni na multidisciplinarne pravice in dolžnosti in ne na proces aplikacije zdravila s strani medicinskih sester.)

Ko govorimo o rokovanju z zdravili, želimo, da pomislite na prispevek posameznega zdravstvenega delavca k optimalni uporabi zdravila in optimalnim izidom zdravljenja.

Na katere dolžnosti pomislite, ko razmišljate o idealni vlogi medicinske sestre v medpklicnem reokovanju z zdravili in kako bi te dolžnosti izgledale v praksi?

Ko razmišljate o teh 4 dolžnostih medicinskih sester, kaj bi radi dodali, spremenili ali odstranili? ***(Pokažemo mu Tabelo 1)***

Tema 2

Prav tako bi želeli definirati naloge, ki bi jih medicinske sestre morale opravljati znotraj teh dolžnosti v idealnem okolju.

Katere specifične naloge naj bi sestra znotraj svoje vloge v multidisciplinarnem timu pri preskrbi pacienta z zdravili po vašem mnenju opravljala?

Kaj bi spremenili, dodali ali odstranili iz podanega seznama nalog medicinske sestre? ***(Pokažemo u Tabelo 2)***

Tema 3

Visoko kakovostna medpoklicno sodelovanje pri rokovanju z zdravili zahteva sporazumno interakcijo med medicinskimi sestrami, zdravniki in farmacevti. Kako bi lahko najbolje opisali idealno medpoklicno sodelovanje med medicinskimi sestrami in ostalimi člani zdravstvenega tima, z namenom doseganja najvišje stopnje kakovosti rokovanja z zdravili?

Kako naj bi po vašem mnenju potekala sodelovanje in komunikacija med medicinskimi sestrami in ostalimi člani zdravstvenega tima, znotraj domene rokovanja z zdravili?

Kaj bi spremenili, dodali ali odstranili s seznama danih interakcij? ***(Pokažemo mu Tabelo 3)***

Tema 4

Sedaj, ko smo predelali dolžnosti, naloge in interakcije medicinske sestre v multidisciplinarnem timu pri rokovanju z zdravili, bi želeli, da premislite o potrebnih predpostavkah, da bi ta idealni model lahko prezrcalili v prakso.

Ob naslednjih vprašanjih imejte v mislih vse omenjene dolžnosti, naloge in interakcije medicinskih sester, navezujoč se na preskrbo pacienta z zdravili.

Katere so prednosti trenutne vloge medicinske sestre pri rokovanju z zdravili?

Katerih nalog/vlog ne bi spreminjali, oziroma za katere mislite, da so dobre v sedanji praksi?

Katere so pomanjkljivosti ali slabosti trenutne vloge medicinske sestre pri rokovanju z zdravili?

Kaj trenutno v praksi ne poteka kot bi moralo?

Kaj bi morali spremeniti znotraj vloge in nalog medicinske sestre, da bi dosegli najvišji nivo kakovosti rokovanja z zdravili?

Kakšne so možnosti za razvoj vloge medicinske sestre pri rokovanju z zdravili, če bi želeli idealni model vloge medicinske sestre pri rokovanju z zdravili prezrcaliti v prakso?

Kaj bi potrebovali za realizacijo te drugačne vloge in kakšne okoliščine bi potrebovali?

Kakšne nevarnosti se lahko pojavijo, če bi model idealne vloge medicinske sestre pri preskrbi pacienta z zdravili poskušali implementirati v dejanske okoliščine?

Kateri faktorji ali okoliščine bi ovirali prenos modela v prakso?

Tabele od 1 do 3

Tabela 1/Dolžnosti

- Nadziranje in spremljanje učinkov zdravila
- Nadziranje in spremljanje učinkovitosti zdravila
- Sprejemanje odločitev o uporabi zdravil, vključujoč predpisovanje zdravil, izključujoč pripravo in administracijo zdravil
- Zdravstveno vzgojno delo in izobraževanje pacientov o uporabi zdravil

Tabela 2/Naloge

- Prepoznavanje sprememb zdravstvenega stanja pacienta in ocena pacientovih potreb
- Registracija zdravila
- Komunikacija v multidisciplinarnem timu (poročanje in opozarjanje)
- Komunikacija s pacientom
- Ukrepanje v primeru nujnih stanj
- Spremljanje učinkov terapije na pacientovo zdravstveno stanje
- Pomoč pri samooskrbi
- »odvisno« predpisovanje zdravil
- »neodvisno« predpisovanje zdravil
- Poročanje o napakah povezanih z medikamentozno terapijo in varnostnih vprašanjih

Tema 3/Interakcije

- Medicinska sestra poroča o opaženem zdravniku in farmacevtu
- Zdravnik zagotovi informacije in navodila medicinskim sestram
- Farmacevt nudi nasvete medicinskim sestram

Interview guide

Spain

Guía de la entrevista para el estudio "Análisis DAFO sobre el papel de las enfermeras en la atención multidisciplinar farmacéutica en Europa: un estudio cualitativo"

Instrucciones basadas en las discusiones durante el programa intensivo, Amberes, 2018:

- El modelo que fue desarrollado de antemano, no se mostrará a los informantes.
- Primero se les pedirá a los informantes que reflexionen de una manera más abierta sobre las responsabilidades, tareas e interacciones de las enfermeras. Luego, les pediremos que reflexionen sobre las cosas que habíamos pensado antes, utilizando los contenidos específicos del modelo relacionados con esa pregunta.
- El entrevistador debe indicar claramente las definiciones de los conceptos utilizados y debe guiar al informante para que comprenda las preguntas y las responda dentro del alcance.
- No se pueden enviar documentos a los informantes de antemano.
- Todos los entrevistadores deben ser transparentes sobre la selección de expertos / casos críticos,aplicando la técnica aprendida en el programa intensivo.
- Todos los datos deben almacenarse de forma segura hasta que el coordinador del proyecto declare explícitamente en papel que los datos se pueden eliminar.
- Cualquier problema durante la recolección de datos o el análisis debe informarse al coordinador del proyecto.
- Le recomendamos que escriba las responsabilidades, tareas e interacciones informadas por los informantes de manera estructurada durante la entrevista, ya que en el tema 2 reflexionaras sobre todas las responsabilidades informadas por los informantes en el tema 1, y en el tema 4 reflexionaras sobre todas las responsabilidades , tareas e interacciones.

Introducción

- Bienvenida al participante: *“Hola, bienvenido. Gracias por estar aquí hoy y por estar dispuesto a participar en esta entrevista ".*
- Describiendo el estudio: *“Esta entrevista es parte de un estudio europeo a gran escala en 14 países sobre el papel de las enfermeras en la atención farmacéutica multidisciplinaria.”*

Se entrevistará a enfermeras, farmacéuticos y médicos de diferentes entornos de atención médica. Nuestro objetivo es desarrollar un modelo general para el papel de las enfermeras en la atención multidisciplinaria farmacéutica, con el fin de mejorar la colaboración multidisciplinaria entre las diferentes profesiones. Queremos que piense cómo sería el papel de las enfermeras en la atención farmacéutica multidisciplinaria en una situación ideal. ¿Qué sería ideal para el cuidado farmacéutico? Luego, le pediremos que reflexione sobre el contexto de la práctica clínica y los requisitos previos para implementar este rol. Lo haremos de forma estructurada preguntando por las fortalezas y debilidades del rol de las enfermeras

hoy en día y sobre las oportunidades y amenazas para el futuro. Nos centraremos en el papel de las enfermeras, no en el papel de otros profesionales de la salud. No hay respuestas buenas o incorrectas a las preguntas. Es importante que dé su opinión personal sobre el tema ".

• *"Puedo pedirle, si es posible, que apague su teléfono móvil".*

• Explicando el curso / procedimiento de la entrevista: “*Esta entrevista será grabada en audio. Después de la entrevista, escribiré la entrevista y analizaré los datos sin su nombre u otros nombres que pueda haber mencionado durante la entrevista. Ahora voy a comenzar la grabación de audio. ¿Le parece bien?*"

• Solicitando el consentimiento informado, ofrece al participante el tiempo necesario para leer el documento completo. *"¿Puedo pedirle que firme un formulario, que se llama 'consentimiento informado'? Esto significa que se le informó sobre el estudio, que tuvo tiempo suficiente para hacer preguntas, que participará de manera voluntaria y sabrá que puede cancelar su participación en cualquier momento sin dar ninguna razón.*

Tema 1

Cuando hablamos de atención farmacéutica, queremos que piense en la contribución de los profesionales de la salud a la atención de las personas para optimizar el uso de medicamentos y mejorar los resultados de salud.

En su opinión, teniendo en cuenta el papel ideal de las enfermeras en la atención multidisciplinaria farmacéutica,

• ¿Qué responsabilidades serían parte del rol ideal de una enfermera? ¿Qué implican estas responsabilidades?

• Al considerar las siguientes 4 responsabilidades, ¿qué le gustaría cambiar, agregar o eliminar?

Responsabilidades:

- monitorización y seguimiento de efectos adversos / terapéuticos,
- monitorización y seguimiento de la adherencia a medicamentos,
- toma de decisiones sobre el uso de medicamentos, incluidos los medicamentos recetados, excluyendo preparación / administración, y
- Proporcionar educación al paciente e información sobre medicamentos.

Tema 2

Dentro de estas responsabilidades, también queremos definir las tareas que las enfermeras realizarían idealmente.

En su opinión, dentro de las responsabilidades previamente definidas de la atención farmacéutica,

• ¿Qué tareas específicas deben desempeñar las enfermeras en su función en el

ámbito multidisciplinario del cuidado farmacéutico?
• Al considerar las próximas tareas, ¿qué te gustaría cambiar, agregar o eliminar?

Tareas:
- Detectar cambios clínicos, problemas de salud o evaluar las necesidades del paciente.
- Registro de medicación
- Comunicación multidisciplinaria (incluyendo informes, alertas y discusión)
- Comunicación con el paciente
- Intervención en caso de emergencia
- Seguimiento
- Apoyo en el autocuidado
- Enfermera "dependiente" que prescribe
- Enfermera "independiente" que prescribe
- Reportar errores de medicación y problemas de seguridad

Tema 3

La atención farmacéutica multidisciplinaria de alta calidad requiere acuerdos sobre la interacción entre enfermeras, médicos y farmacéuticos. ¿Cómo podemos describir mejor la colaboración y comunicación ideal entre las enfermeras y otros profesionales de la salud, con el objetivo de obtener una atención farmacéutica de alta calidad?

En su opinión, dentro del ámbito de la atención farmacéutica,
• ¿En qué cree que debería consistir la colaboración y la comunicación entre las enfermeras y otros proveedores de atención médica en la atención farmacéutica multidisciplinaria?
• Al considerar las siguientes interacciones, ¿qué te gustaría cambiar, agregar o eliminar?

Interacciones:
- Enfermeras reportando observaciones a médicos y farmacéuticos.
- Médicos que proporcionan información e indicaciones a las enfermeras.
- Farmacéuticos asesorando a las enfermeras.

Tema 4
Hemos discutido sobre las responsabilidades de las enfermeras, las tareas y las interacciones multidisciplinares en la atención farmacéutica multidisciplinaria. Nos gustaría que reflexionemos ahora sobre los requisitos previos para hacer realidad este modelo ideal.

Por favor considere todas las responsabilidades, tareas e interacciones multidisciplinares.
1. ¿Cuáles son las fortalezas del papel de las enfermeras en la atención farmacéutica multidisciplinaria? ¿Qué está funcionando bien ya? ¿Qué te gustaría mantener?
2. ¿Cuáles son las debilidades del rol de las enfermeras en la atención farmacéutica multidisciplinaria? ¿Qué no está funcionando en este momento? ¿Qué se debe cambiar en el rol de las enfermeras para lograr una atención farmacéutica de alta calidad?
3. Si quisiéramos poner en práctica el modelo ideal del rol de las enfermeras en la atención farmacéutica multidisciplinaria, ¿cuáles son las oportunidades? ¿Qué hace que sea más fácil realizar la implementación? ¿Cuáles son las circunstancias favorables?
4. Si quisiéramos poner en práctica el modelo ideal del papel de las enfermeras en la atención farmacéutica multidisciplinaria, ¿cuáles son las amenazas? ¿Qué hace más difícil realizar la implementación? ¿Cuáles son las circunstancias desfavorables?
