# Supplementary material for: Perspectives of nurses’ role in interprofessional pharmaceutical care across 14 European countries: A qualitative study in pharmacists, physicians and nurses
Source: PLoS One. 2021 May 27;16(5):e0251982. doi: 10.1371/journal.pone.0251982 (PMC8158867; doi:10.1371/journal.pone.0251982)
Supplement: S2 Appendix — (PDF) [file pone.0251982.s002.pdf]

Contribution to the study	Names of contributors (country)
Data collection (interviewer)	Hilde Feyen (Belgium)<br>Tina Geets (Belgium)<br>Ellen Verstrepen (Belgium)<br>Lucie Polakova (Czech Republic)<br>Věra Nigrovičová (Czech Republic)<br>Martina Hašková (Czech Republic)<br>Petra Vilímová (Czech Republic)<br>Marion Baltes (Germany)<br>Juliane Friedrichs (Germany)<br>Thomas Klatt (Germany)<br>Panayiota Kalatzi (Greece)<br>Maria Iliadou (Greece)<br>Galata Aggeliki (Greece)<br>Miklós Sugár (Hungary)<br>Éva Horváth (Hungary)<br>Anita Lelovics (Hungary)<br>Marika Lo Monaco (Italy)<br>Tiziana Avenoso (Italy)<br>Daniela Stefanova (North-Macedonia)<br>Biljana Markovska (North-Macedonia)<br>Angela Parmakovska Trifunovski (North-Macedonia)<br>Siepke van den Burg (The Netherlands)<br>Remco Verbrugge (The Netherlands)<br>Lidia van Veenendaal (The Netherlands)<br>Carina Marie Nome (Norway)<br>Tina Reinertsen Aardalen (Norway)<br>Julie Stenbjerg (Norway)<br>Marie Helen Arnesen (Norway)<br>Ellen Marie Bjørling (Norway)<br>José Miguel Sousa Pedro Seguro (Portugal)<br>Susana Simões Calhindro (Portugal)<br>Inês Simões Pereira (Portugal)<br>Darina Farkašová (Slovakia)<br>Barbora Belcáková (Slovakia)<br>Patrícia Sontagová (Slovakia)<br>Benjamin Osmancevic (Slovenia)<br>Mojca Čolnik (Slovenia)<br>Marko Petrovic (Slovenia)<br>Alfredo Pérez Marco (Spain)<br>Jorge Riquelme-Galindo (Spain)<br>Adrián Marco (Spain)<br>Lisa Matthews (UK)<br>Sarah Keeley (UK)<br>Kevin Akerman (UK)
Data analysis, interpretation of data, review of manuscript	Zuzana Kafkova (Slovakia)<br>Igor Karnjuš (Slovenia)
Facilitating access to the field	Bence L. Raposa (Hungary)<br>Rønnaug Larsen (Norway)

	Håkon Johansen (Norway)<br>Christin Slydahl (Norway)
Other contribution	Katerina Chvojkova (Czech Republic)<br>Kalafati Maria (Greece)<br>Alberto De Santis (Italy)<br>Giuseppe Andrea De Biase (Italy)<br>Maria Isabel Domingues Fernandes (Portugal)<br>Amélia Filomena de Oliveira Mendes Castilho (Portugal)<br>Paulo Alexandre Carvalho Ferreira (Portugal)
