# Supplementary material for: Perspectives of nurses’ role in interprofessional pharmaceutical care across 14 European countries: A qualitative study in pharmacists, physicians and nurses
Source: PLoS One. 2021 May 27;16(5):e0251982. doi: 10.1371/journal.pone.0251982 (PMC8158867; doi:10.1371/journal.pone.0251982)
Supplement: S1 Table — Codes S001 to S042 were the codes of the first code book, codes S043 to S049 were added to the final code book. (PDF) [file pone.0251982.s004.pdf]

Themes	Code
drug monitoring	M01
monitoring adherence	M02
prescribing medicines	M03
patient education	M04
pharmaceutical care in general, not specified	M05
interprofessional collaboration	M06
pharmaceutical model implementation	M07
strenghts nurses' role in interprofessional PC	M08
weaknesses of nurses' role in interprofessional PC	M09
opportunities of nurses' role in interprofessional PC	M10
threats of nurses' role in interprofessional PC	M11

Sub-themes	Code	Short description / clarification of the sub-theme
active involvement in research	S001	Clinical research for highly educated nurses
added value of nurse	S002	Characteristics of nurses and nursing which make the contribution of nurses of added value to the contributions of other professionals in pharmaceutical care
administrative tasks	S003	Administrative tasks of nurses in PC
availability of professionals	S004	Professionals available for other professionals or for patients
care coordination	S005	The coordination of different steps in the care for patients. Interprofessional pharmaceutical care is one system, like a chain.
collaboration between professions	S006	Collaboration between nurse-physician, nurse-pharmacist, pharmacist-physician, nurse-nurse. Interprofessional collaboration.
communication with patient/ informal caregiver	S007	Communication with patient/ informal caregiver

communication with professionals	S008	Mono-disciplinary or multidisciplinary communication, nurses questioning physicians, physicians questioning nurses, communication with individuals or with the entire staff. Written or oral communication.
competences	S009	Nurses' competences in different situations, concerning different tasks/responsibilities, resistance against certain competences. Competences, including knowledge, attitudes, skills and abilities
confidence	S010	Trust in/from other professionals, trust of patients in health care professionals
current nursing practice in pharmaceutical care	S011	Common practice for nurses, tasks that they are already performing currently
decision-making	S012	Making decisions about pharmaceutical care related topic
definition of pharmaceutical care	S013	Definition of pharmaceutical care
detecting clinical change / healthcare problems / assessing patient needs	S014	Detecting clinical change / healthcare problems / assessing patient needs
differentiation in function between different levels of nursing	S015	According to different levels of education. E.g. Specialised nurses.
equality - hierarchy	S016	Nurses are equal to physicians and pharmacists in interprofessional collaboration/communication versus there exists a hierarchy between the professions
(financial) rewards and recognition system	S017	Wages of nurses relative to their responsibilities. Recognition of their competences. Aknowledge nurse qualifications.
follow-up	S018	Follow-up adverse/therapeutic effects, follow-up of clinical change/patient status
independent nurse consultation	S019	Nurses can be consulted independently from a physician.
intervention in case of emergency	S020	Nurse interventions in case of emergency
laws and regulations	S021	Legal framework
necessity of change in interprofessional pharmaceutical care	S022	Is changing the current situation necessary? Urgent or not.
nurse advise to other professional	S023	A nurse giving advice to other health professionals
nurse close to patient	S024	Availability of nurses for patients, their (constant) presence on the floor, leading to the nurses having more information about the patient.

nurse education	S025	Level of nurse education - extending or adjusting nurse education to allow nurses being involved in different aspects of pharmaceutical care - vocational education, extra education, mandatory (or not) education
nurse receiving advise from other professional	S026	Nurse receiving advise from other professional
patient characteristics	S027	Patient characteristics can influence the involvement of nurses in pharmaceutical care
patient needs	S028	Needs and expectations of patients and informal caregivers
patient safety	S029	(Improving) patient outcomes, safe pharmaceutical care. Safety of current nurse involvement or increase of nurse involvement. Risks, medication errors and safety issues.
pharmacists' role	S030	Not the aim of this study, but pharmacists' role can also be mentioned in the interviews.
physicians' role	S031	Not the aim of this study, but physicians' role can also be mentioned in the interviews.
prerequisites	S032	Requirements, conditions, necessities for nurse involvement in pharmaceutical care, prerequisites for the model to be implemented
quality of care	S033	The levels of excellence which characterize the health service or health care provided based on accepted standards of quality
registration	S034	Registration and documentation in patient records
reporting	S035	Reporting to other professionals
representation of physician in case of absence	S036	A nurse represents a physician in case of absence or in case of awaiting a physician
responsibility	S037	Responsibilities of nurses in pharmaceutical care, being responsible for a task or for a person
self-care support	S038	A nurse supporting self-care of patients is a task of nurses in pharmaceutical care
sense of responsibility	S039	Nurses feeling responsible towards pharmaceutical care, patients, …
setting of employment	S040	In which setting are nurses employed
shortage of nurses	S041	Shortage of nurses in different health care settings and in health care in general
supervision	S042	Supervision of newly admitted, supervision of students
culture	S043	Ward culture of hospital department
ICT support	S044	Pharmaceutical care, using technology, software, web applications, patient platforms, …
nurses' role	S045	Nurses’ role in interprofessional pharmaceutical care
patient’s freedom of choice	S046	Freedom of choice when selecting healthcare professionals
therapy reconciliation	S047	The process of ensuring that two sets of medication therapy (eg. Medication list at home versus in hospital) are in agreement

turn-over	S048	Intention to leave and turn-over of nurses
workload	S049	Nurses’ workload and patients per nurse ratio, time to care
